# Advancing Antibiotic-Resistant Microbe Combat: Nanocarrier-Based Systems in Combination Therapy Targeting Quorum Sensing

**DOI:** 10.3390/pharmaceutics16091160

**Published:** 2024-09-03

**Authors:** Sanchaita Rajkhowa, Safrina Zeenat Hussain, Manisha Agarwal, Alaiha Zaheen, Sami A. Al-Hussain, Magdi E. A. Zaki

**Affiliations:** 1 Centre for Biotechnology and Bioinformatics, Dibrugarh University, Dibrugarh 786004, Assam, India; safrinazeenatk18@gmail.com (S.Z.H.); manishaagarwal1022@gmail.com (M.A.); izaheen03@gmail.com (A.Z.); 2 Department of Chemistry, Imam Mohammad Ibn Saud Islamic University (IMSIU), Riyadh 11623, Saudi Arabia; sahussain@imamu.edu.sa

**Keywords:** quorum sensing, quorum quenching, biofilm, quorum quenching inhibitors, nanocarriers

## Abstract

The increase in antibiotic-resistant bacteria presents a significant risk to worldwide public health, emphasizing the necessity of novel approaches to address infections. Quorum sensing, an essential method of communication among bacteria, controls activities like the formation of biofilms, the production of virulence factors, and the synthesis of secondary metabolites according to the number of individuals in the population. Quorum quenching, which interferes with these processes, emerges as a vital approach to diminish bacterial virulence and prevent biofilm formation. Nanocarriers, characterized by their small size, high surface-area-to-volume ratio, and modifiable surface chemistry, offer a versatile platform for the disruption of bacterial communication by targeting various stages within the quorum sensing pathway. These features allow nanocarriers to infiltrate biofilms, disrupt cell membranes, and inhibit bacterial proliferation, presenting a promising alternative to traditional antibiotics. Integrating nanocarrier-based systems into combination therapies provides a multi-pronged approach to infection control, enhancing both the efficacy and specificity of treatment regimens. Nonetheless, challenges related to the stability, safety, and clinical effectiveness of nanomaterial-based antimicrobial treatments remain. Continued research and development are essential to overcoming these obstacles and fully harnessing the potential of nano-antimicrobial therapies. This review emphasizes the importance of quorum sensing in bacterial behavior and highlights the transformative potential of nanotechnology in advancing antimicrobial treatments, offering innovative solutions to combat antibiotic-resistant pathogens.

## 1. Introduction

Bacteria, as fundamental unicellular entities, possess the inherent capability to grow, divide, and respond to environmental cues independently. Despite their inherent autonomy, bacteria exhibit coordinated behaviors with neighboring cells to perform collaborative tasks. This coordination is orchestrated through a complex mechanism known as quorum sensing (QS), where bacterial cells communicate to collectively regulate gene expression in response to population density fluctuations. QS underlies the production of key virulence factors essential for bacterial pathogenesis and supports the formation of biofilms and cohesive communities that adhere to surfaces [1,2,3].

Biofilm formation and QS are tightly connected processes in microbial communities. Biofilm formation entails the collective behavior of bacterial populations embedded within a self-produced extracellular matrix. A cell-to-cell communication mechanism, known as QS, modulates gene expression in response to the population density. Traditionally, it was thought that QS might prompt a shift to a biofilm lifestyle once a specific population density is reached. However, evidence from various bacterial species suggests otherwise. QS activation occurs within an established biofilm, coordinating both maturation and breakdown. This reveals a dynamic interaction between QS and biofilm development, where QS governs not only the initiation but also the progression and disassembly of biofilms [4].

**Figure 1:**
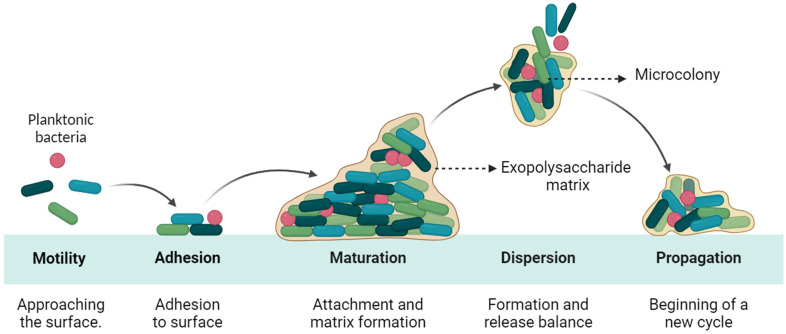
**Biofilm life cycle (createdin © BioRender—biorender.com).** The biofilm formation process consists of three main stages: the initial irreversible attachment to a surface, bacterial proliferation accompanied by the synthesis of an extracellular matrix, and the breakdown of the matrix, leading to bacterial dispersal. This schematic depicts the formation and progression of biofilms, emphasizing the protective role of the extracellular matrix and the durability of the bacterial communities within these structures.

Biofilm formation is characterized by the aggregation of microorganisms adhered to surfaces or interfaces ensconced within a self-generated extracellular matrix. Within this structured community, bacteria thrive and are shielded from various environmental adversities, including desiccation, immune system assaults, predation by protozoa, and antimicrobial substances. The mechanistic understanding of biofilm formation involves three distinct phases: the initial irreversible attachment to the substrate, followed by bacterial proliferation and the synthesis of an extracellular matrix, and concluding with the degradation of the matrix, resulting in bacterial dispersal. Figure 1 displays a schematic representation illustrating the formation of a biofilm [5].

Biofilms pose a significant threat to living organisms and constitute over 80% of human infections, presenting a significant challenge in treatment owing to the inherent resistance of sessile bacteria. Biofilm-related infections result in more than 500,000 fatalities each year, with treatment expenses estimated at approximately USD 94 billion [6]. Sessile bacteria within biofilms demonstrate antibiotic resistance levels that are increased up to a thousandfold compared to their planktonic counterparts. Additionally, their reduced exposure to the host immune system adds to the complexity of therapeutic interventions [7]. The increase in antibiotic resistance may be attributed to the enhanced spread of resistance genes, the activity of efflux pumps, and the acquisition of resistant traits. Additionally, biofilms demonstrate highly dynamic structural properties and rapid shifts in gene expression, which result in modifications to their surface antigens [8,9]. A fully matured biofilm poses formidable challenges in eradication owing to its robustness and resistance, particularly against antibiotic interventions. The high concentration of metal ions and the low pH within the biofilm establish an environment that hinders diffusion, thereby reducing the efficacy of antibiotics. This environment facilitates antibiotic inactivation, ultimately aiding bacterial cells within the biofilm to withstand treatment [10]. The effective eradication of a fully developed biofilm necessitates the utilization of compounds capable of penetrating its intricate structure or mechanically disrupting its integrity. The persistent difficulty in biofilm removal, coupled with escalating antibiotic resistance, underscores the urgency of exploring novel strategies to counteract undesirable microbial populations. Among these strategies, targeting the QS system holds promise as a prospective intervention avenue.

The rise in antimicrobial resistance and the increasing prevalence of infectious diseases pose significant challenges to the medical community, necessitating the urgent development of novel therapeutic approaches. Addressing these pressing concerns requires innovative, long-term solutions. An extensive array of quorum quenching (QQ) compounds has recently been discovered. Some studies have shown encouraging therapeutic benefits when utilized alongside conventional antimicrobial medications. However, clinical trials have revealed a low success rate, primarily due to the poor stability, biocompatibility, absorption, and delivery to target sites of these QSIs [11,12]. Consequently, researchers are actively seeking improved solutions to address these challenges. 

Recent advancements in nanomaterials have led to the development of a new generation that surpasses the functionality of traditional bulk materials. These nanomaterials are distinguished by their high reactivity and large surface-area-to-volume ratio, which facilitate the precise tuning of their physicochemical properties. This has made them highly attractive for various biomedical applications, including their promising role in combating pathogenic biofilms. Innovative strategies have emerged, such as the integration of peptides, metallic polymer composites, and hybrid systems, as well as the use of antibiotic-encapsulated nanoparticles and responsive smart nanomaterials. These approaches aim to harness the antimicrobial properties while minimizing the adverse effects on the host. The use of drug-loaded nanoparticles offers a solution to the common challenges associated with traditional treatments, such as toxicity, inadequate delivery, and enzymatic degradation [13,14]. Recent studies have demonstrated that various nanostructures possess strong antimicrobial properties, offering promising potential for the treatment of infectious diseases [15]. Nanosized particles present several strategic benefits compared to conventional antimicrobials, including enhanced efficacy, reduced toxicity, minimized resistance development, and cost-effectiveness. Advanced nanodrug delivery systems have been engineered to deliver antibiotics with high precision, ensuring prolonged cellular or tissue retention and sustained therapeutic effects. These nanodrug carriers address the challenges inherent to traditional antibiotic therapies, such as limited bioavailability and rapid clearance. As a result, nanomaterials have emerged as promising alternatives in treating bacterial infections. Their nanoscale operation allows for the precise control of antimicrobial activity, paving the way for improved strategies against multidrug-resistant pathogens and enhanced clinical outcomes [16,17].

This review provides a comprehensive analysis of bacterial biofilms, QS, and QQ via enzymatic and natural compounds, alongside a thorough overview of nano-enabled strategies to combat biofilm-associated infections. It emphasizes the synergistic potential of nanocarrier-based systems when combined with QS-targeted therapies, showcasing their ability to surpass the limitations of traditional treatments. The review also explores the latest advancements in nanocarriers, highlighting their role in enhancing drug delivery, minimizing side effects, and improving the efficacy of QS inhibitors.

## 2. QS: Communication Processes in the Formation of Bacterial Biofilms

Cell communication and signaling play pivotal roles in the development and growth of multicellular organisms. Initially, it was believed that such coordination was exclusive to eukaryotes, with bacterial interactions presumed to be indirect and driven mainly by shifts in nutrient availability. However, recent advancements over the past two decades have revealed that bacteria employ intricate communication systems to regulate diverse biological processes, including the development of biofilm communities and the modulation of virulence factors [18].

At low densities, bacteria exhibit characteristics typical of single-celled organisms. However, upon reaching a critical population density, they transition to multicellular-like behavior. This transition is regulated by QS, a process where intercellular signaling molecules called autoinducers influence gene expression based on the population density. QS requires a critical threshold in terms of both the bacterial number and signal concentration to induce differential gene regulation. This regulatory system facilitates the coordination of complex bacterial behaviors, including the formation of biofilms, the secretion of virulence factors, competence acquisition, sporulation, bioluminescence, and secondary metabolite production, within their communities [19].

QS mechanisms are crucial regulators of virulence in both Gram-positive and Gram-negative bacteria. QS involves a signaling cascade where diffusible molecules interact with transcriptional regulators, either directly or through the activation of sensor kinases. This process begins with bacterial cells synthesizing small signaling molecules that are subsequently released into the environment. Upon reaching a critical concentration, these molecules bind to specific receptors, triggering changes in gene expression. A common outcome of QS-mediated gene expression is the increased production of proteins involved in signaling molecule synthesis. This increased protein production triggers a positive feedback loop, leading to quorum signals being commonly termed autoinducers [20].

Bacterial QS involves a wide range of signaling molecules, including oligopeptides, Autoinducer-2 (AI-2), N-acyl homoserine lactones (AHLs), hydroxy-palmitic acid methyl ester, and methyl dodecanoic acid. Among these, AHLs and peptide-based signals are the most extensively studied. AHLs, primarily produced by Gram-negative bacteria, diffuse through the cell membrane and interact with intracellular regulatory proteins. In contrast, peptide-based QS in Gram-positive bacteria typically involves membrane-bound receptor histidine kinases [21,22,23,24]. Additionally, there exists a third type of QS signal, known as AI-2, that is present in both bacterial types. It has garnered attention as a potential target for the control of multispecies infections [25].

### 2.1. QS in Gram-Negative Bacteria

**Figure 2:**
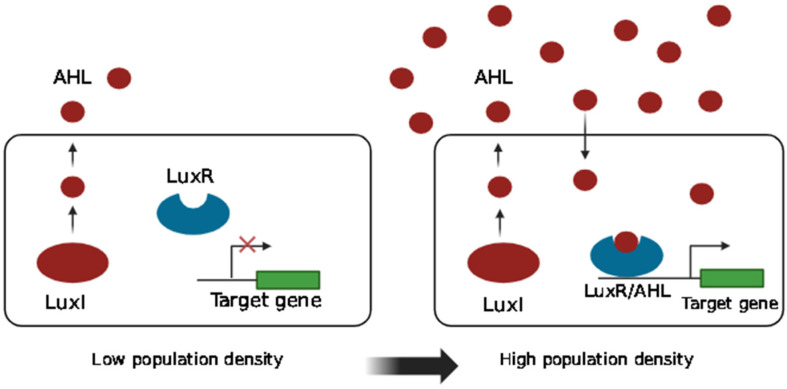
**Mechanism of QS mediated by AHLs in Gram-negative bacteria.** AHL signals originate from autoinducer synthases within the LuxI family. Under conditions of low population density, the AHL levels remain minimal, resulting in the rapid deterioration of the unstable LuxR receptor. Once the AHL concentration surpasses a certain threshold, it binds to LuxR and initiates its activation. The AHL/LuxR complex subsequently modulates the transcription of targeted genes. The figure illustrates how Gram-negative bacteria use AHLs for QS, highlighting the versatility of AHL production and recognition among different species.

The lux-type QS system plays a critical role in species-specific communication among Gram-negative bacteria, utilizing the production and detection of AHLs. This system generally consists of two primary components: an autoinducer synthase (such as LuxI), which generates AHLs from S-adenosylmethionine, and a transcriptional regulator (such as LuxR) that mediates the response to these signals [20]. Most Gram-negative bacteria that use QS rely on LuxI family autoinducer synthases to generate QS signals characterized by a homoserine lactone core with a variable side chain. The specificity of these signals is determined by differences in the side chain of the AHL, including variations in the chain length (ranging from 4 to 18 carbons), substituent R groups, and degree of saturation [26]. Owing to its small size and lipophilic nature, the AHL diffuses freely across cell membranes. As the population density rises, the intracellular AHL binds to its cognate LuxR-like receptor in the cytoplasm, leading to differential gene expression. The AHL/LuxR complex, once activated, proceeds to engage with responsive promoters, regulating downstream gene transcription. Figure 2 illustrates how Gram-negative bacteria engage in AHL-mediated QS. The production and recognition of AHLs highlight their remarkable versatility, with individual bacterial species capable of synthesizing multiple AHL types and distinct AHLs being detected by various bacterial species. For example, *Pseudomonas aeruginosa* produces different homoserine lactone (HSL) QS molecules, such as C4-HSL and 3-oxo-C12-HSL via the autoinducer synthases RhlI and LasI, respectively, to regulate gene expression and facilitate intercellular communication. Conversely, various bacteria, including *Pantoea ananatis*, *Edwardsiella tarda*, *Rhizobium leguminosarum*, *Erwinia psidii*, and *Serratia marcescens*, produce C7-HSL [26,27].

### 2.2. QS in Gram-Positive Bacteria

**Figure 3:**
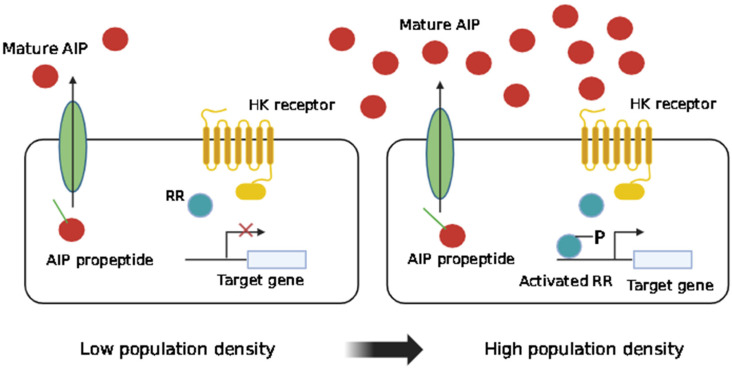
**QS in Gram-positive bacteria facilitated by AIPs.** AIPs emerge as propeptides initially and typically undergo post-translational modifications before being secreted by specialized transporters. Upon achieving elevated concentrations, the AIP binds to and activates the specific transmembrane histidine kinase (HK) receptor. This activation subsequently triggers a response regulator (RR). The activated RR then modulates the transcription of target genes.

Gram-positive pathogens utilize intercellular communication to modulate virulence through mechanisms that differ from those of Gram-negative bacteria. Unlike Gram-negative bacteria, which use AHLs or quinolones as signaling molecules, Gram-positive bacteria rely on modified oligopeptides known as autoinducing peptides (AIPs) for this regulatory process [20]. Translationally derived AIPs serve as QS signals in numerous Gram-positive bacteria. These peptides frequently undergo post-translational modifications, such as the geranylation of tryptophan residues (e.g., ComX in *Bacillus subtilis*) or the formation of a thiolactone ring (e.g., AgrD in *Staphylococcus aureus*) [1,28]. Their secretion frequently requires specialized transport machinery for proteins. Upon release, transmembrane histidine kinases detect AIPs, initiating a two-component signal transduction system. This system comprises a histidine kinase and a cytoplasmic response regulator, with phosphorelay mechanisms facilitating signal transmission. The phosphorylated response regulator then modulates transcription from specific promoters, triggering a cascade of transcriptional events that ultimately result in changes in gene expression [29,30]. Figure 3 depicts how Gram-positive bacteria engage in AIP-mediated QS.

### 2.3. Alternative Forms of QS

AI-2 is a signaling molecule regulated by the LuxS enzyme, present in both Gram-negative and Gram-positive bacteria. This molecule assesses the density and diversity of microbial populations in the surrounding environment, facilitating appropriate metabolic adjustments. Originally discovered in the bioluminescent system of *Vibrio harveyi*, the LuxS/AI-2-mediated QS system involves the synthesis of AI-1 and AI-2 signaling molecules. AI-1, controlled by the LuxLM gene, interacts with its receptor, LuxN, while AI-2, a distinct signaling molecule, engages with its receptors, LuxP and LuxQ. Signal transduction is facilitated by LuxU, a phosphotransferase that transmits phosphorylation signals to LuxO, a regulatory protein. Phosphorylated LuxU subsequently interacts with LuxR to initiate the expression of target genes. The pathogenicity of *Edwardsiella tarda* has been linked to the LuxS/AI-2-mediated QS system, underscoring its critical role in modulating virulence factors and pathogenic behavior [31,32].

## 3. QQ: Nature’s Answer to Antibiotic Resistance

**Figure 4:**
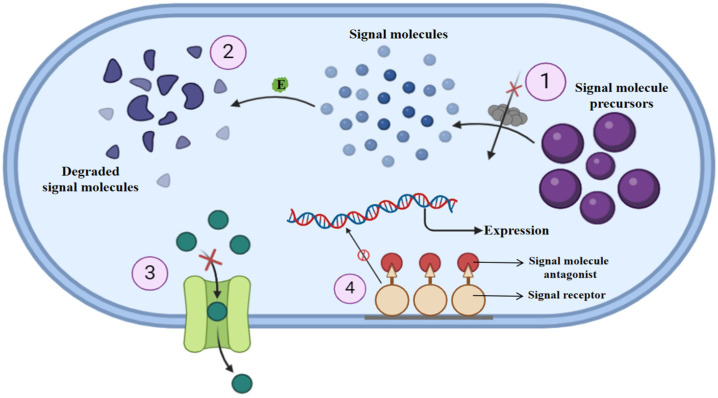
**Schematic representation of QQ techniques employed to inhibit bacterial QS** (**created in © BioRender—biorender.com).** 1. Direct suppression of the synthesis of signaling molecules. 2. Chemical or biological degradation of signaling molecules. 3. Disruption of the transport mechanisms of signaling molecules. 4. Competitive inhibition that obstructs the binding of signaling molecules to their receptors.

The rising resistance of microorganisms to conventional antibacterial agents presents a significant challenge in medical practice. Even newly developed drugs, once believed capable of overcoming resistance, are now showing diminished efficacy against certain bacterial strains. This underscores the urgent need for innovative antimicrobial strategies that can effectively target resistant microorganisms while maintaining long-term efficacy. Recent research has pivoted towards targeting specific bacterial components involved in virulence factor production, marking a shift from the traditional approach of inhibiting bacterial growth. A promising method in this domain is QQ or quorum quenching. QQ obstructs biofilm formation and inhibits the synthesis of virulence factors by attenuating QS, a critical communication mechanism within bacterial communities. QQ disrupts bacterial communication through four key mechanisms. First, it inhibits the biosynthesis of QS signal molecules by blocking the enzymatic pathways responsible for their production, thus preventing the initiation of essential signaling activities. Second, QQ hampers the transport of these signal molecules across bacterial membranes, impeding their spread within and between microbial communities and disrupting coordinated behaviors. Third, QQ involves the degradation of QS signals, either chemically or biologically, neutralizing them before they can interact with target receptors. Lastly, QQ employs competitive inhibition, where QQ molecules mimic natural QS signals and bind to receptors without triggering activation. This prevents the natural signal molecules from binding, effectively blocking the downstream signaling pathways critical for bacterial cooperation and pathogenicity [33,34,35,36]. Figure 4 depicts the mechanisms by which the QS system is inhibited. The pathways involve disrupting signal molecule synthesis, degrading or inactivating signaling molecules, interfering with signal reception, and inhibiting QS-regulated gene expression. 

QQ is particularly attractive because of its non-lethal effects on pathogens and its capacity to alleviate severe selective pressures, which diminishes the likelihood of drug resistance development [37]. Consequently, QQ emerges as a promising strategy for biological control, offering potential as a novel approach to antibacterial treatment and the management of microbial populations.

QQ encompasses a spectrum of processes aimed at disrupting QS, which is a pivotal mechanism governing microbial communication and biofilm formation. These processes involve the modulation of QS signals and the disruption of multiple components, including extracellular DNA, exopolysaccharides, proteins, lipopolysaccharides, and secondary messengers. Collectively, these mechanisms impede biofilm formation [38]. The inherent architectural stability and resilience of biofilms, coupled with their heightened resistance transmission rates and low permeability to antibiotics, present formidable hurdles for conventional antibiotic therapies. Addressing these challenges often necessitates employing mechanical, physical, and chemical strategies for biofilm disruption [39]. Frequently employed strategies for the elimination of microbial biofilms consist of adhesion inhibitors, QQ molecules, exopolysaccharide disruptors, and competitive microorganisms. Moreover, natural or synthetic substances formulated to affect the QS system, referred to as QS inhibitors (QSI), function through the QQ mechanism. These inhibitors offer promising avenues to combat biofilm-associated infections by interfering with microbial communication and by impeding biofilm formation [40,41,42,43].

QSIs can be categorized into three main groups. The first group involves enzymes that degrade autoinducers by chemical modification. Enzymes such as lactonases, reductases, amidases, and cytochrome oxidases are present in a variety of microorganisms, including bacteria, fungi, archaea, marine species, and other organisms [44]. The second category includes organic substances found in nature, such as phenolic compounds like flavonoids, tannins, and phenylpropene, as well as indole derivatives, furanones, alkaloids, organosulfur compounds, lactones, and acetaldehydes [33,45]. The third group comprises artificial derivatives of QS compounds, which encompass AHL derivatives (for example, azithromycin, macrolides, furanyl hydrazide, and cyclohexanone) and lactone derivatives (such as N-(heptyl-sulfanyl acetyl)-L-HSL, 4-phenylbutanoyl-HSL, and HepS-AHL) [19]. Furthermore, natural substances, chelating agents, synthetic analogs, and antibiotics are employed to prevent the growth of biofilms. The treatment approach commonly involves using a combination of antibiotics to combat bacterial infections caused by complex biofilms comprising multiple species. Moreover, antimicrobial peptides, whether naturally existing or produced via genetic manipulation (like the wide-ranging bactericidal peptide R-FV-I16), have demonstrated effectiveness in breaking down biofilms [46].

## 4. QQ Agents: A Source for Anti-Virulence Treatment

QQ agents are compounds or substances that interfere with QS mechanisms in bacteria. The disruption of QS has garnered considerable attention in various fields because of its potential to control bacterial behavior without killing them. Researchers have identified a wide array of QQ agents sourced from diverse organisms and environments, demonstrating the potential for novel treatments derived from natural and synthetic compounds [47,48]. QQ agents can be categorized into various groups depending on how they work.

### 4.1. Natural QSIs

QSIs function by competitively impeding microbial QS systems, thereby interrupting the complex signaling cascades essential for coordinating intra- and inter-species communication in the expression of virulence factors [49]. In natural ecosystems, diverse organisms cohabit, engaging in intricate interactions. It is widely recognized that bacteria rely on communication mechanisms to both collaborate within their own populations and contend with others. Within this framework, competition ensues through the quenching of QS systems via an array of compounds [19].

#### 4.1.1. QSIs Derived from Animal Sources

Interactions between eukaryotic hosts and pathogenic bacteria elicit a diverse range of responses, notably modulated by QS molecules. In plant hosts, responses to QS signals such as AHLs include mechanisms such as the production of AHL decoy molecules, enhanced root growth, increased stomatal activity, and the uptake of these signals into the shoots [50,51,52]. QQ enzymes have been identified in various animal models, including mice, zebrafish, and rats. For instance, porcine kidney acylase I has been demonstrated to inactivate QS signals such as N-Hexanoyl-L-Homoserine Lactone (C6HSL) and 3-Oxo-Dodecanoyl-L-Homoserine Lactone (3OC12HSL), while N-Butanoyl-L-Homoserine Lactone (C4HSL) remains unaffected [23]. Acylase I demonstrates a moderate level of efficacy in the suppression of biofilm formation by bacteria such as *Aeromonas hydrophila* and *Pseudomonas putida* [53]. Mammalian paraoxonases (PONs) are known for their hydrolytic activity on esters and lactones, playing a crucial role in drug metabolism and nerve agent detoxification [54]. PON-lactonases are distinct from their prokaryotic homologs due to the absence of the “HCDH-H-D” motif and their requirement for calcium ions to function. Additionally, human epithelial cells have been shown to degrade AHLs produced by *Pseudomonas aeruginosa*, with the efficiency of degradation being influenced by the length of the acyl chain [55,56]. Serum from various mammals, including bovine, goat, rabbit, horse, and mouse serum, has demonstrated the ability to deactivate the QS signal 3OC12HSL [57]. Numerous studies have documented the presence of AI-2 molecules in certain food items, with some demonstrating AI-2-inhibitory properties [58]. The presence of AI-2 in food products may arise from microbial sources or from compounds within the food that mimic the activity of the AI-2 autoinducer [59]. Inhibitory impacts on AI-2 function have been observed in a range of items, such as turkey patties, chicken breast, artisanal cheeses, beef steaks, and beef patties [60]. In 2004, Lu and collaborators illustrated that a range of meat items, such as ground beef and poultry, possess the ability to impede AI-2 activity, which was assessed using a *Vibrio harveyi* reporter strain. Extracts from ground beef were identified to hinder AI-2-mediated bioluminescence in *V. harveyi*, resulting in the modification of the expression of genes associated with virulence. This suggests that compounds within ground beef may have therapeutic potential by disrupting AI-2 sensing pathways. Specifically, these compounds interfere with classical AI-2 sensing and counteract AI-2-induced gene expression changes [61]. Table 1 summarizes the QSIs derived from various animal sources, outlining their origins, their target organisms, and the QS activities that they modulate.

#### 4.1.2. QSIs Derived from Fungi

Fungi are well known for their production of secondary metabolites, including antibiotics. *Penicillium* spp., in particular, have been a significant source of antibiotics such as penicillin, renowned for combating bacterial infections for nearly a century [62]. Recent research has pinpointed roughly 33 *Penicillium* spp. capable of synthesizing QSIs like patulin and penicillic acid [63]. In a study using a mouse model of pulmonary infection, the use of patulin was found to be highly effective in reducing infections caused by *Pseudomonas aeruginosa*. Additionally, certain fungi found in the plant rhizosphere, particularly those belonging to the Ascomycota and Basidiomycota phyla, have shown the ability to break down C6HSL and 3-Oxo-Hexanoyl-L-Homoserine Lactone (3OC6HSL) through their lactonase activity [64]. Additionally, natural pigments produced by *Auricularia auricularis* have been observed to possess QSI properties, effectively inhibiting the production of violacein in *Chromobacterium violaceum* [65]. Table 2 provides an overview of QSIs from different fungi sources, detailing their active components, their target organisms, and the specific QS activities that they influence.

#### 4.1.3. QSIs Derived from Marine Organisms

Marine organisms serve as abundant reservoirs of QSIs that interfere with bacterial communication mechanisms. Among these organisms, marine cyanobacteria in particular contain unique compounds with notable biological activity and structural distinctiveness, displaying considerable potential for biomedical and pharmaceutical applications. Marine cyanobacteria display remarkable diversity in AHL-dependent QSIs, indicating their potential role in regulating microbial communities by disrupting intercellular communication pathways. For instance, *Blennothrix cantharidosmum*, a marine cyanobacterium, generates tumonoic acids that efficiently inhibit the bioluminescent function of *Vibrio harveyi* BB120 without impeding the growth of the bacteria [67]. Furthermore, *Lyngbya majuscula*, a marine cyanobacterium, produces a range of compounds, including lyngbyoic acid, malyngolide, 8-epi-malyngamide C, and lyngbic acid. These compounds effectively inhibit the LasR receptor in *Pseudomonas aeruginosa*, counteracting the effects of exogenous 3OC12-HSL [68]. Of particular interest are honaucins derived from *Leptolyngbya crossbyana*, which exhibit inhibitory effects on both QS and inflammation processes [69]. This multifunctional activity suggests a new approach to developing therapeutics with broad-spectrum efficacy.

The microorganism known as *Halobacillus salinus* generates two chemical compounds, namely N-(2-phenylethyl)-isobutyramide and 3-methyl-N-(2-phenylethyl)-butyramide, that have the ability to impede the production of violacein in *Chromobacterium violaceum* CV026 in the presence of AHLs [70]. Both *Bacillus cereus* and *Marinobacter* sp. SK-3 produce diketopiperazines (DKPs) that impede AHL-mediated QS pathways [71]. Moreover, piericidin, a compound obtained from actinobacteria found in the marine environment, hinders the process of violacein production in the bacterium *Chromobacterium violaceum* strain CV026 [72]. Marine algae, notably *Delisea pulchra*, produce halogenated furanones with significant antifouling and antimicrobial properties. These furanones are thought to interact with AHL receptors due to their structural resemblance to AHLs [73,74]. Furanones have the capacity to interfere with the activity of the AHL-triggered transcriptional activator LuxR in *Vibrio fischeri*, leading to a reduction in LuxR’s ability to attach to DNA in *Vibrio harveyi*. Moreover, furanones possess the capability to influence the AI-2 pathway present in both Gram-negative and Gram-positive bacteria by chemically altering and subsequently deactivating the AI-2 synthase LuxS [75,76]. Halogenated furanones have protective effects against *Vibrio* species in rotifers, shrimp, rainbow trout, and mice, although some furanones are toxic to certain organisms and human fibroblasts [77,78,79]. Furthermore, a combination of betonicine, floridoside, and isethionic acid produced by *Ahnfeltiopsis flabelliformes* inhibits QS-dependent responses in the *Agrobacterium tumefaciens* NTL4 (pCF218) (pCF372) reporter strain, specifically affecting 3OC6-HSL activity [80]. Table 3 provides an overview of the QSIs derived from different marine organisms, detailing their active components, their target organisms, and the specific QS activities that they influence.

#### 4.1.4. QSIs Derived from Plants

Numerous sources of health-promoting foods and traditional remedies, including fruits, herbs, and medicinal plants, have been identified for their potential to yield QSI. Nonetheless, only a limited number of these compounds have undergone isolation or detailed structural and biochemical characterization. Within this group, a variety of phenolic compounds have demonstrated the ability to inhibit AHL-dependent QS pathways. The QSI properties found in plant extracts are linked to their structural similarity to AHLs, as well as their capability to break down signal receptors like LuxR/LasR [89,90]. For instance, γ-aminobutyric acid (GABA), synthesized by certain plant species, promotes the degradation of the AHL signal (OHC8HSL) through the action of the AttM lactonase enzyme derived from *Agrobacterium tumefaciens*, thus attenuating the infection process that is reliant on QS [91]. Pyrogallol, obtained from therapeutic botanical sources such as *Emblica officinalis*, along with its structural derivatives, demonstrates inhibitory properties against AI-2 [92]. The liberation of L-canavanine from *Medicago sativa* seeds exerts a significant impact on the expression of QS, particularly modulating the synthesis of exopolysaccharides by *Sinorhizobium meliloti*, a bacterium renowned for its nitrogen-fixing capabilities [93]. Seedlings of *Medicago truncatula* influence the activity of CviR, AhyR, and LuxR reporter systems in multiple species, significantly affecting QS, particularly in *Pseudomonas aeruginosa* and *Sinorhizobium meliloti* [94].

Cinnamaldehyde and its by-products influence a broad spectrum of QS-mediated activities, including biofilm formation in *Pseudomonas aeruginosa* and AI-2 mediated QS in various *Vibrio* spp. [95]. Furocoumarins, naturally present in grapefruit, have been shown to inhibit the activity of *Vibrio harveyi* reporter strains BB886 and BB170 by targeting both AI-1 and AI-2 signaling pathways. Additionally, these compounds suppress biofilm formation in *Escherichia coli* O157:H7, *Salmonella typhimurium*, and *Pseudomonas aeruginosa* [96]. The seeds of sour oranges harbor limonoids, chemical compounds that exhibit robust inhibitory effects on AI-2 activity within *Vibrio harveyi* bacteria [97].

Flavonoids are ubiquitous compounds found in plants and plant-derived products, including propolis and honey. They demonstrate a diverse array of pharmacological effects across various biological systems, indicating their potential utility in a broad spectrum of therapeutic applications, and they possess diverse chemical structures [98]. An illustrative example is catechin, a flavan-3-ol extracted from the bark of *Combretum albiflorum*. This flavonoid is the first of its kind, recognized for its ability to reduce the synthesis of virulence factors by interfering with the function of the RhlR regulator in *Pseudomonas aeruginosa* PAO1 [99]. Honey and propolis have demonstrated inhibitory effects on QS mechanisms in *Chromobacterium violaceum* and *Pseudomonas aeruginosa*, respectively. These effects are likely attributable to the abundant presence of flavonoids within these substances [98]. Flavonoids have attracted considerable scientific interest due to their established anti-inflammatory, antioxidant, and potential anticancer effects. In particular, flavonoids like kaempferol, naringenin, quercetin, and apigenin are being explored for their potential as QSIs given these beneficial properties. The flavonoids investigated here showed the significant inhibition of luminescence expression driven by HAI-1/AI-2 in *Vibrio harveyi* strains MM32 and BB886. Notably, naringenin and quercetin were highly effective in reducing biofilm formation by *Vibrio harveyi* strain BB120 and *Escherichia coli* O157:H7 [100].

Various leguminous plants, like peas, alfalfa, clover, and yam beans, have exhibited the capacity to degrade AHLs, potentially through lactonase enzymatic activity, although the precise enzyme responsible for this function awaits complete elucidation [101]. *Arabidopsis* exudates were also observed to influence QS signals in *Agrobacterium tumefaciens* [102]. Aqueous extracts derived from *Ananas comosus*, *Musa paradisiaca*, *Ocimum sanctum*, and *Manilkara zapota* have demonstrated QSI characteristics. These extracts effectively hindered violacein production by *Chromobacterium violaceum*, a commonly used indicator of inhibition. Additionally, they exhibited inhibitory effects on various virulence factors associated with *Pseudomonas aeruginosa* PAO1, including pyocyanin pigment production, staphylolytic protease and elastase production, and the formation of biofilms [103]. Additionally, the essential oil and hydrosol of *Satureja thymbra*, along with polytoxinol—a compound derived from the essential oil—demonstrated significant efficacy in inhibiting biofilm formation by *Staphylococcus*, *Lactobacillus*, *Pseudomonas*, *Listeria*, and *Salmonella* spp. [104]. Table 4 provides a comprehensive overview of the QSIs derived from various plant sources. It highlights the diversity of natural plant compounds and their potential in modulating QS pathways across different bacterial species.

### 4.2. Macromolecules as QSIs

Various macromolecular agents have demonstrated the ability to interfere with QS processes. Unlike small-molecule QSIs, these macromolecular QQ agents primarily function by catalyzing the degradation of signaling molecules, rather than competing with signal receptors. Although the majority of macromolecular QQ agents primarily target AHL-dependent QS pathways, enzymatic degradation has also been documented for *Pseudomonas* quinolone signal (PQS), diffusible signal factor (DSF), and AI-2 signaling molecules. Notably, bacterial strains such as *Bacillus*, *Staphylococcus*, and *Pseudomonas* exhibit enzymatic activity that quenches DSF [115]. Present investigations in the realm of macromolecular QQ compounds primarily focus on QQ enzymes that exhibit the capacity to degrade AHL signaling molecules. Moreover, certain research findings suggest that specific antibodies have the capacity to impede QS mechanisms by sequestering or enzymatically degrading AHL signaling molecules [36]. AHL-degrading enzymes are classified into three main categories based on their enzymatic functions: AHL lactonases, which catalyze lactone hydrolysis; AHL acylases, which promote amide bond hydrolysis; and AHL oxidases and reductases, which facilitate oxidation–reduction reactions. AHL lactonases hydrolyze the ester bonds in AHL molecules, converting them into N-acyl-homoserine. AHL acylases cleave the amide bond, producing homoserine lactone and the corresponding fatty acid. AHL oxidases and reductases alter AHL structures through redox reactions. Typically, AHL lactonases need metal ions to function and can act on AHLs with diverse acyl chain lengths. In contrast, acylases show specificity depending on the acyl chain length and β-position substitutions of the AHL chain [48,116]. Table 5 lists the sources of macromolecular QSIs, the enzymes involved, and the specific QS signal molecules that they degrade.

#### 4.2.1. AHL Lactonases

AHL lactonases are enzymes that hydrolyze the homoserine lactone rings within AHL molecules, producing acyl homoserine. This enzymatic function is not exclusive to bacteria but has also been identified in eukaryotic cells. Initially detected in *Bacillus* species, AHL lactonase activity has now been reported in around 30 different taxa. These enzymes are categorized into several families, notably the phosphotriesterase (PTE) family and metallo-β-lactamase superfamily. The metallo-β-lactamase superfamily, specifically, has undergone extensive examination and is widely distributed across various bacterial taxa. Within this superfamily, specific clusters, including AidC, AiiA, and a recently discovered marine AHL lactonase cluster, have been delineated [117]. The first AHL lactonase to undergo characterization, AiiA derived from *Bacillus* sp. 240B1, is classified within the metallo-β-lactamase family. At the outset, it was posited that the substrate specificity of AiiA was predominantly oriented towards the amide bond present in homoserine lactone along with its associated acyl side chain. However, recent studies have revealed that AiiA predominantly cleaves the ester bond. Structurally, AiiA shares features with the metallo-hydrolase superfamily, including a conserved motif, “His^104^-X-His^106^-X-Asp^108^-His^109^”, similar to the zinc-binding motifs in various metalloenzymes. Functional analyses, including site-directed mutagenesis, have highlighted a critical motif, “His^106^-X-Asp^108^-His^109^-59X-His^169^-21X-Asp^191^”, that is essential for AHL lactonase activity. AiiA demonstrates broad substrate specificity, favoring AHLs with longer acyl chains. The manifestation of AiiA in *Burkholderia thailandensis*, *Pseudomonas aeruginosa*, and *Erwinia carotovora* results in the significant reduction of AHL levels and the suppression of virulence gene expression. These findings suggest that AiiA could be a promising therapeutic target in reducing bacterial virulence [118].

In addition to metallo-β-lactamases, a distinct class of AHL lactonases known as PTE-like lactonases (PLLs) shares notable sequence and structural similarity with PTEs, particularly in the architectures of their active sites. PLLs are widely distributed across bacterial and archaeal domains. Although PLLs lack significant sequence homology with AiiA, they exhibit variable AHL-degrading capabilities and utilize metal ions for their catalytic functions. Notably, PLLs generally show lower AHL-degrading efficiency compared to AiiA. Additionally, all identified PLLs exhibit broad-spectrum phosphotriesterase activity. Bacterial PTEs, classified within the amidohydrolase superfamily—a heterogeneous group of hydrolytic enzymes with varied functions—are likely evolved from ancestral PLLs, exploiting their inherent paraoxonase activity [119,120,121,122].

The activity of AHL lactonases has been observed across a range of eukaryotic organisms, including those found in mammalian serum. Unlike bacterial lactonases, serum lactonases require calcium ions for their enzymatic function, underscoring their characteristics as paraoxonases with lactonase capabilities. Notably, the paraoxonases Pon1, Pon2, and Pon3 play crucial roles in AHL degradation in mammalian serum. These enzymes have been demonstrated to disrupt QS in *Pseudomonas aeruginosa* biofilms, indicating their potential to influence bacterial communication mechanisms [123,124].

#### 4.2.2. AHL Acylases

Acylase enzymes from the AHL acylase family, classified under the QQ group, effectively hydrolyze amide bonds in AHLs, resulting in the formation of homoserine lactones and fatty acids. This irreversible degradation positions AHL acylases as highly effective QSIs with extensive practical applications. *Variovorax paradoxus* was the first bacterium to exhibit AHL acylase activity [125]. These enzymes have been identified in various bacterial genera, including *Arthrobacter*, *Pseudomonas*, *Streptomyces*, *Ochrobactrum*, *Nostoc*, and *Brucella*. In contrast to AHL lactonases, which are associated with diverse protein families, the majority of the identified AHL acylases are classified within a singular superfamily distinguished by an N-terminal nucleophile (Ntn) hydrolase structural configuration. This structural motif is characteristic of beta-lactam acylases, including penicillin G acylase [126]. The AHL acylases classified within the Ntn hydrolase superfamily can be divided into two discrete clusters, namely AAC and QuiP. Evidence suggests that the QuiP cluster may possess a greater capacity for AHL degradation when compared to the AAC cluster. Certain constituents of the QuiP cluster exhibit the capacity to degrade AHLs as short as C6-HSL or even C4-HSL, whereas members of the AAC cluster generally degrade AHLs with chain lengths that surpass C8-HSL [48].

#### 4.2.3. AHL Oxidoreductases

To date, oxidative and reductive AHLase activities have been identified solely in bacterial species. Unlike traditional QQ enzymes, these AHLases do not degrade AHLs into inactive forms. Instead, they modify the chemical structures of the AHL signals. This modification does not directly lead to the degradation of AHLs; however, it may influence the distinctiveness and identification of the AHL signal, thereby potentially hindering processes regulated by QS [127]. The initial occurrence of bacterial AHL catabolic activity was identified in *Rhodococcus erythropolis*. AHLs possessing 3-oxo substituents undergo expedited degradation by means of the reduction of the keto group located at the β position, culminating in the generation of 3-hydroxy-derivative AHLs [64]. The second AHL oxidoreductase, CYP102A1 from *Bacillus megaterium*, a well-characterized cytochrome P450 enzyme, oxidizes AHLs at the ω-1, ω-2, and ω-3 positions of the acyl chain. This enzyme also shows significant activity with ring-opened AHLs and fatty acid chains, which are products of AHL lactonase and acylase actions, respectively [127]. BpiB09, the third oxidoreductase identified, is an NADH-dependent enzyme discovered through metagenomic analysis. When expressed in *Pseudomonas aeruginosa*, BpiB09 led to decreased swimming motility, reduced pyocyanin production, and impaired biofilm formation, ultimately diminishing the bacterium’s pathogenicity against *Caenorhabditis elegans* [128].

**Table 5:** Macromolecular QSIs sourced from bacteria.

Enzyme	Source Organism	Degraded QS Signal Molecule	Reference
Lactonase	*Bacillus* sp. strain 240B1	AHLs	[118]
Lactonase	*Bacillus thuringiensis*	AHLs	[129]
Lactonase	*Oceanobacillus* strains 30, 172, and 97-2	AHLs	[130]
Lactonase	*Halomonas* sp. strain 33	AHLs	[130]
Lactonase	*Acinetobacter* sp. strain C1010	AHLs	[131]
Acylase/Lactonase	*Tenacibaculum discolor* strain 20J	AHLs	[130]
Acylase/Lactonase	*Hyphamonas* sp. DG895	C4HSL and 3OC12-HSL	[130]
Acylase	*Alteromonas* sp. strain 168	C4HSL and 3OC12-HSL	[130]
AHL Acylase	*Bacillus pumilus* S8-07	3OC12-HSL	[132]
AHL Acylase	*Ralstonia* sp. XJ12B	Long-chain AHLs	[133]
AHL Acylase	*Pseudomonas aeruginosa* PAO1	Long-chain AHLs	[134]
AHL Lactonase	*Rhodococcus erythropolis* strain W2	AHLs	[135]
AHL Lactonase	*Agrobacterium tumefaciens*	AHLs	[136]
AHL Lactonase	*Arthrobacter* sp. IBN110	AHLs	[137]
AHL Oxidoreductase	*Burkholderia* strain GG4	3OC6-HSL	[138]

## 5. Nanotechnology: A Promising Strategy against Multidrug-Resistant Bacteria

The anti-virulence potential of QQ enzymes and small-molecule QSIs has been rigorously validated in both in vitro and in vivo settings. Because each technique has different molecular characteristics and modes of action, it has different advantages and limits.

QSIs primarily target specific signal receptors or receptor families, such as the LuxR-like family, providing an avenue for the development of drugs aimed at inhibiting the expression of virulence factors in pathogens. However, microbial infections often involve multiple pathogenic species, and a single drug may not suffice to combat such complex infections. On the other hand, a variety of AHLs can be broken down by QQ enzymes, especially AHL lactonases, which may make them more effective in combating polymicrobial infections. Nonetheless, the broad specificity of AHL-degrading enzymes raises concerns about possible unintended effects, such as disrupting beneficial AHL-dependent QS activities in the host microbiota.

QSIs, characterized by their simpler molecular structures, are amenable to synthetic modification and provide precise temporal control over biological systems due to their rapid diffusion. Their non-proteinaceous nature and low molecular weight also reduce the risk of eliciting antibody-mediated immune responses, a concern with protein-based QQ enzymes. However, stability remains a challenge for both QSIs and QQ enzymes. QSIs, especially those structurally similar to native AHLs, are prone to degradation by environmental QQ enzymes, while QQ enzymes are susceptible to proteolytic degradation and thermal instability [139,140,141].

Resistance to QQ compounds has emerged as a notable challenge, raising doubts about the suitability of QS as a target for anti-virulence therapy. Experiments involving the culture of *Pseudomonas aeruginosa* PA14 with adenosine as the sole carbon source and exposure to the synthetic QSI furanone C-30 have shown this resistance [142]. Despite the potential efficacy of anti-virulence drugs, none of them have been brought to the market yet, partially because there are no appropriate delivery systems available. Furthermore, the effectiveness of these drugs depends on accurate pathogen diagnosis for the selection of appropriate compounds, underscoring the importance of fostering the advancement of diagnostic tools to inform treatment decisions [143]. In this context, advanced nanotechnology offers substantial benefits for biofilm inhibition. The deployment of nanomaterials, particularly nanoparticles (NPs), has been harnessed to enhance the bioavailability, selectivity, and stability of active compounds while mitigating their toxicity. 

A minimum of one dimension smaller than 100 nm characterizes NPs, making them the cornerstone of nanotechnology. In the medical field, NPs are envisioned as precise tools for targeted drug delivery, ensuring optimal concentrations and durations at specific sites—an approach termed “nanomedicine”. Over the past few decades, extensive research has focused on nanotechnology’s medical applications, particularly in drug delivery. The rising prevalence of infectious diseases and the limitations of current antimicrobial therapies present significant challenges, necessitating innovative solutions. Nanomedicine offers a promising strategy by utilizing engineered materials within this nanoscale to develop novel therapeutic and diagnostic approaches. Recent advancements in nanomaterials have garnered significant attention due to their enhanced drug functionalities, surpassing conventional materials. Bridging materials science and nanobiotechnology, these nanomaterials exhibit superior physical, chemical, and biological properties, positioning them as a promising avenue for the development of effective antimicrobial therapies. Emerging research highlights various nanostructures with potent antimicrobial properties, holding significant potential in treating infectious diseases [144]. 

The application of biocompatible and biodegradable NPs in targeted gene or drug delivery has emerged as a highly effective therapeutic approach. Enhancing the distribution of anti-infective agents represents a relatively recent advancement in drug delivery, particularly within the realm of nanomedicine. These NPs have unique interactions with biological systems, high surface-area-to-mass ratios, enhanced reactivity, and nanoscale dimensions, among other distinctive physicochemical features. When drugs are incorporated into NPs using techniques like physical encapsulation, adsorption, or chemical conjugation, their pharmacokinetics and therapeutic efficacy are significantly improved over traditional drug formulations [145].

The importance of metals like copper (Cu), zinc (Zn), titanium (Ti), gold (Au), and silver (Ag) has been highlighted by recent advancements in nanotechnology. These metals have been used therapeutically due to their broad-spectrum activity against a variety of microorganisms, with NPs demonstrating antimicrobial properties and gaining significant scientific recognition as potent inhibitory agents against pathogen growth [146]. Recently, researchers have begun to apply nanotechnology to the creation of sophisticated nanoantibiotics. These nanoantibiotics have a number of benefits, including improved mucus and biofilm penetration, increased solubility, sustained QSI activity, and efficient delivery. Furthermore, the application of nanofactories consisting of nanobiomolecules, nanobiostructures, nanobiohybrids, and nanozymes has led to the generation of novel antimicrobials with the ability to modify bacterial QS systems [147].

In the domain of antibacterial therapy formulation, it is essential to devise carriers that stabilize active ingredients and enhance their delivery to infection sites. Nanocarrier-based drug delivery systems enable precise targeting and enhanced therapeutic efficacy, thereby overcoming the limitations associated with conventional QSIs. These systems facilitate the targeted delivery of QSIs, ensuring higher local concentrations at the infection site and thereby achieving the more effective disruption of biofilms and QS mechanisms. Many nanoscale delivery systems, including lipid–polymer hybrid nanoformulations, PLGA NPs, and fusogenic liposomes, have also shown promise in the past 20 years as a means of delivering medications to infection sites [148,149].

## 6. Exploration of Nanotechnological Strategies

Understanding the antibacterial processes of NPs has become a major area of study because of the increased use of nanomaterials in biomedical domains [150]. NPs have the ability to modify the metabolic activity of bacteria via a range of interactions, including van der Waals forces, hydrophobic interactions, electrostatic attraction, and receptor–ligand interactions [151]. They can also cross the bacterial membrane and aggregate along metabolic pathways, changing both the shape and function of the cell membrane. Lastly, NPs affect the fundamental components of bacterial cells, causing oxidative stress, changes in their permeability and gene expression, imbalances in electrolyte levels, the inactivation of proteins, and the inhibition of enzymes [152]. 

Nanospheres (solid framework) and nanocapsules (liquid core cavity surrounded by a wall) are two different forms of NPs. Only nanospheres have been thoroughly investigated for use in the administration of antibiotics. Obtaining efficient loading is less possible with highly polar chemicals, because ordinary polymers are hydrophobic. Alkyl cyanoacrylate monomers are emulsion-polymerized with medication to create classical NPs. Nucleophilic agents cause this reaction. The anionic polymerization of the monomer may occasionally be induced by the antimicrobial agent. A covalent bond between the drug and polymer may cause the drug to become partially or completely unavailable. As a result, it is essential to continuously compare the drug’s in vitro antibacterial activity in NP form to that of the free drug. The pace at which antibiotics are released from NPs and the rate at which esterases break down the polymer have been found to be substantially linked. As a result, in an esterase-free solution, the release from NPs is minimal; however, when carboxyesterase is present, it increases significantly. Lysosomal esterases can destroy colloidal carriers that have been endocytosed by phagocytic cells within endosomes. Generally, the use of monomers with long side chains increases the entrapment efficiency [153].

**Figure 5:**
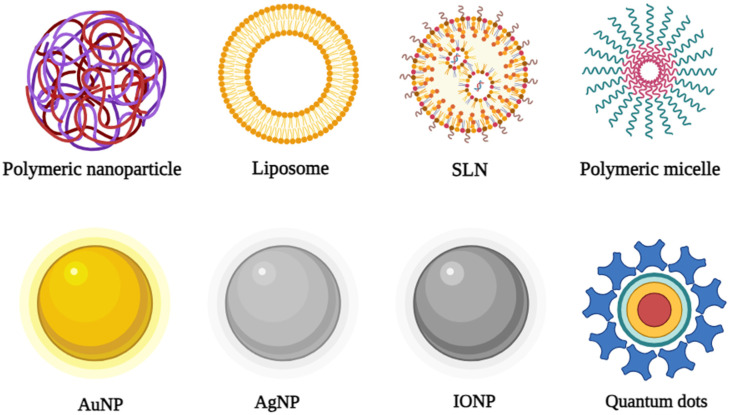
**Examples of NP classes (created in © BioRender—biorender.com).** The NP classes depicted in this figure encompass organic NPs such as polymeric nanoparticles, liposomes, solid–lipid nanoparticles (SLNs), and polymeric micelles, alongside inorganic NPs including gold nanoparticles (AuNPs), silver nanoparticles (AgNPs), iron oxide nanoparticles (IONPs), and quantum dots.

NPs can be either inorganic or organic depending on their intended use. Figure 5 summarizes some of the NP classes. Organic NPs encompass a variety of structures, such as SLNs, polymeric micelles, polymeric NPs, and liposomes, which are extensively used in therapeutic applications [154]. They offer significant advantages, such as the capability to handle both hydrophobic and hydrophilic drugs, compatibility, biodegradability, and minimal systemic toxicity. Nonetheless, they possess various drawbacks, such as a brief shelf life, inadequate encapsulation effectiveness, instability at high temperatures, and vulnerability to severe processing circumstances. Huh et al. provided an extensive overview of organic NPs, including their use in combination therapies and controlled release [155].

Metallic NPs, on the other hand, have remarkable stability, high loading capacities, and small sizes. These metallic NPs are engineered at the atomic level using nanotechnology, enabling them to exhibit size-dependent properties that are conducive to various applications in catalysis, biotechnology, medicine, and pharmaceuticals. They act as drug delivery systems and therapeutic agents and aid in medical diagnostic imaging [156].

Because of their high surface–volume ratio, inorganic NPs have unique physicochemical properties (chemical, optical, electronic, magnetic, mechanical, and catalytic). This enables them to surpass barriers and exhibit enhanced activity in biological systems when compared to bulk materials. In particular, metallic NPs show promise as antibacterial agents, circumventing the limitations associated with antibiotics and bulk metals. They are extensively used in drug and gene delivery, radiotherapy, diagnostic assays, and thermal ablation techniques [157].

### 6.1. Organic NPs

The most emphasis has been placed on organic NPs, among the other categories. These include SLNs, polymeric micelles, liposomes, and polymeric NPs. Their outstanding compatibility and biodegradability are their main features [158]. Systemic toxicity is reduced because NPs readily break down into physiologically acceptable compounds that are metabolized through the Krebs cycle. They are also capable of carrying both hydrophilic and hydrophobic drugs [159]. However, they have shortcomings, such as poor loading efficiency, medicine loss in storage and transit, and relatively low stability. Moreover, they are prone to opsonization and rapid deterioration owing to their hydrophilic/hydrophobic properties [160]. The characteristics, including the descriptions, advantages, limitations, and toxicity profiles, of organic NPs are comprehensively summarized in Table 6.

### 6.2. Inorganic NPs

Inorganic NPs consist of various oxides that differ in shape, solubility, long-term stability, and size. They are produced through the reduction of metallic salts (e.g., gold, silica, titanium, silver, and aluminum) using a reducing agent [170]. The characteristics of these NPs can be tailored based on the reaction conditions, such as the reducing agent concentration, pH, temperature, or reduction time [171]. The selection of a reducing agent plays a critical role in drug delivery, influencing parameters such as NP aggregation, antimicrobial activity, loading capacity, and drug release. In comparison to organic NPs, metallic NPs offer a smaller size and increased loading capacity, with some possessing intrinsic antimicrobial properties [172]. Inorganic NPs primarily accumulate in the liver, spleen, and lymph nodes, making them especially appropriate for the treatment of reticuloendothelial system-related illnesses. However, they are prone to accumulation, metabolism, and aggregation. Usually, the body eliminates substances through bile, a saturable and slow process, potentially leading to prolonged accumulation. Nonetheless, the accumulation rate varies depending on the particular metal used, because some metals have well-established physiological absorption and excretion mechanisms [173].

Furthermore, increased red blood cell changes, spleen weight, inflammation, and liver or skin damage can be caused by the interaction of inorganic NPs with proteins. Intracellularly, both healthy and infected cells can experience cytotoxicity, immunotoxicity, and genotoxicity owing to the production of harmful ions, as well as the estimation of metallic NPs in these tissue types. However, the toxicity can be mitigated by reducing the NP concentrations of inflammatory cytokines and reactive oxygen species (ROS). Chronic toxicity, such as nephrotoxicity, pulmonary toxicity, or hepatotoxicity, may arise from their accumulation or from the implementation of chemical transformations such as surface modifications or changes in size [174]. Table 7 outlines the descriptions, advantages, limitations, and toxicity of inorganic NPs, highlighting their distinct properties and how they differ from their organic counterparts.

## 7. Combination Therapies: Integrating Small Molecules and Nanotechnology Approaches

Combination treatments that target many pathways in bacterial pathogens provide a potential strategy to counteract antibiotic resistance. Combining small molecules with nanotechnology offers a versatile approach, in which the former can improve medication administration and bioavailability, while the latter can impair bacterial signaling pathways. In addition to addressing the drawbacks of traditional antibiotics, this synergistic method uses the special qualities of nanomaterials to increase treatment efficacy, reduce adverse effects, and circumvent bacterial resistance mechanisms.

### 7.1. Nanocarriers for Antibacterial Agents Produced by Plants

Owing to the growing knowledge of the adverse consequences of synthetic preservatives and conventional antimicrobials, the incorporation of naturally occurring substances with antimicrobial activity is gaining considerable interest [16]. Essential oils have remarkable antibacterial properties that enable them to effectively combat harmful microbes. Essential oils are aromatic, viscous liquids derived from several plant compounds and are recognized for their therapeutic benefits [187]. Such types of substances are readily oxidized to produce chemicals that are less biologically active or cause hypersensitivity. As these oils can maintain therapeutic medication blood levels by enhancing the solubility, stability, and bioefficacy of formulations based on essential oils, it has been proposed that these oils can be nanoencapsulated and used in drug delivery systems. Numerous studies have shown how effective essential oils produced with nanotechnology are as antimicrobials [16]. Carvacrol and eugenol, the two main phenolic components of clove and oregano essential oils, respectively, demonstrated higher antibacterial activity in the NP form than in their free forms. One well-known benefit of polymer nanocapsules is their simple inclusion into gels or creams, resulting in the creation of innovative biopharmaceuticals and cosmeceuticals. The antimicrobial activity of thymol-loaded methylcellulose/ethyl cellulose polymeric NPs prevented the growth of *Staphylococcus aureus*, *Pseudomonas aeruginosa*, and *Escherichia coli* in both oil/water lotion and hydrophilic gel conditions [188].

Lipid NPs consist of lipids in addition to phospholipids, which enable these carriers to interact with a range of cells, in addition to serving as an appropriate medium for the trapping of essential oils [189]. In order to improve the stability, solubility, and distribution of lipophilic antibiotics for the treatment of microbial infections, these nanomaterials can be employed as carriers [16]. Several encapsulated terpenes’ antibacterial properties have been explored [190]. In accordance with Cristani et al., [191] by dissolving the lipid component of the *S. aureus* cell membrane, NPs of lipids containing thymol and menthol exhibit more toxicity to the bacteria than their free counterparts. Several variables can be related to the synergistic effect. First, thymol and menthol are released gradually and under control by the NPs, giving the bacterial cells extended exposure. Second, better penetration into the bacterial cell membrane is facilitated by lipid-based NPs, which makes it possible to distribute the active ingredients more effectively. Furthermore, thymol and menthol could work in concert to enhance several cellular functions in the bacterium, intensifying their harmful effects. Overall, the increased bactericidal activity against *S. aureus* is the consequence of the synergistic relationship between the lipid NPs and the encapsulated thymol and menthol, demonstrating the promise of this nanomaterial-based strategy in treating bacterial infections.

The antibacterial activities of *Origanum dictamnus*, *Thymus* spp., and *Zanthoxylum tingoassuiba* essential oils have been shown to be superior in nanoencapsulated formulations compared to their free forms [191]. Insights from the formulation of essential oils as nanoemulsions have also been encouraging. A sunflower oil nanoemulsion showed favorable antibacterial efficacy against several pathogenic bacteria, including *Staphylococcus aureus*, *Pseudomonas aeruginosa*, and *Escherichia coli* [26]. A variety of cooking oils were assessed for their ability to produce a surfactin-based nanoemulsion, including sunflower, sesame, peanut, coconut, and castor oils. When compared to surfactin-based emulsions, the sunflower oil-based nanoemulsion exhibited the lowest drop size, ranging from 72.52 to 875.22 nm. The sunflower oil-based nanoemulsion, known as AUSN-1, also had the greatest antibacterial activity against *Listeria monocytogenes*, *Staphylococcus aureus*, and *Salmonella typhi*. AUSN-1 also had substantial fungicidal action against *Aspergillus niger*, *Rhizopus nigricans*, and *Penicillium* spp., in addition to a considerable sporicidal ability against *Bacillus cereus* and *Bacillus circulans*. A significant decrease in naturally existing cultivable bacterial and fungal populations was seen when AUSN-1’s antimicrobial activity was evaluated under real-world settings in food products such as uncooked chicken, mixed vegetables, milk, and apple juice. The nanoencapsulation of NPs is a potential approach to the administration of lipophilic natural compounds.

### 7.2. NPs Derived from Plants

Plant secondary metabolism, which is greatly impacted by environmental factors, produces natural products from a variety of plant organs and tissue types, such as the roots, seeds, bark, flowers, stalks, and shoots, and this process can be readily accelerated by external signals [192,193]. Since plant-based NPs have a high degree of stability and can be produced quickly, the process of the biosynthesis of metallic NPs has generated interest regarding the elucidation and characterization of the mechanisms involved in the absorption and bioreduction of metal ions by plants [194]. As a result, a large body of research has demonstrated that the presence of various secondary metabolites, such as coenzymes, phenolic substances, steroids, flavonoids, terpenoids, alkaloids, tannins, and saponins, in plant extracts makes them potentially safe precursors for the synthesis of nanomaterials. Additionally, these metabolites function as stabilizing and reducing agents throughout the bioreduction process that yields metallic nanoparticles [192,195]. Thus, a variety of bio-based NPs, including those based on zinc oxide, palladium, platinum, gold, and silver, as well as magnetite, cobalt, and copper, have been effectively produced using plants. In the process of the biosynthesis of NPs, extracts from a variety of species of plants have also been used [196]. Certain plants, such as *Helianthus annuus* (sunflower, Asteraceae), *Medicago sativa* (alfalfa, Fabaceae), and *Brassica juncea* (mustard greens, Brassicaceae), can collect considerable quantities of silver when metals are present in the substrate. Living plants have also been used to synthesize Ni, Co, Zn, and Cu NPs [197,198].

**Figure 6:**
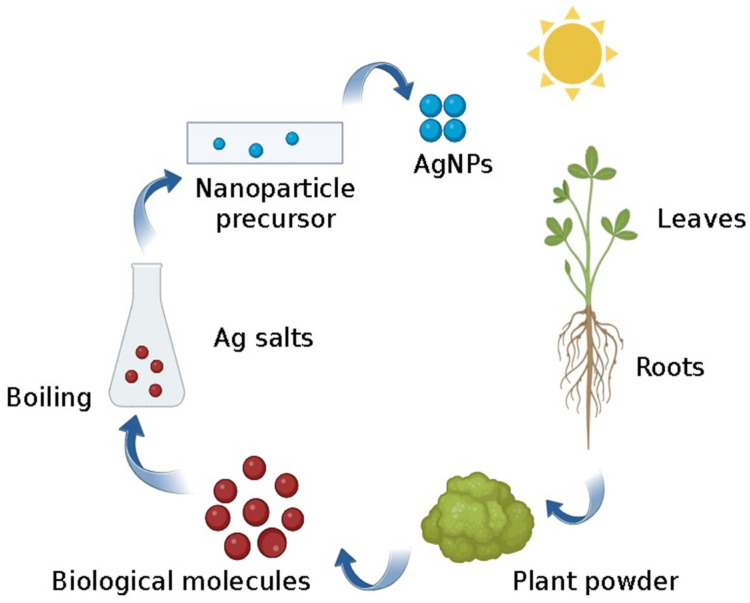
**Synthesis of NPs from plant extracts (created in © BioRender—biorender.com).** Plant secondary metabolism, influenced by environmental factors, produces natural products from various plant tissue types and organs, accelerating the biosynthesis of metal NPs. Plant extracts rich in secondary metabolites like phenolics, alkaloids, and flavonoids serve as effective precursors for diverse bio-based NPs, including silver, gold, and zinc oxide.

Interestingly, plant-generated NPs have a wider range of forms and sizes than those produced by other organisms, indicating that their size and shape are heavily influenced by their location. Therefore, when acquiring NPs gathered within a single plant, it is crucial to recognize that those from other plants may vary depending on the specific plant part from which they are extracted [199]. According to Zhang et al. [200], AgNPs derived from green synthesis exhibited directly proportional antibacterial and antifungal effects to their concentrations, whereas AgNPs derived from fungi demonstrated superior antifungal characteristics against *Pityrosporum ovale* and *Candida albicans*, two types of superficial mycoses [201]. They may also be used to break down methylene blue dye [202]. For instance, a tuber extract from *Curcuma longa* (Zingiberaceae) exhibited the higher synthesis of AgNPs than the powder, which was explained by the extract’s high concentration and readily accessible levels of water-soluble reducing agents, which were primarily responsible for reducing the silver ions in the AgNPs [203]. It has also been reported that AgNPs may be synthesized quickly—in only five hours—by reducing aqueous Ag^+^ ions with a tuber extract of *Dioscorea bulbifera* (Dioscoreaceae). Strong antibacterial action against Gram-positive as well as Gram-negative bacteria was demonstrated by the resultant AgNPs. Owing to its distinct phytochemistry, this plant species has extensive medicinal applications [204]. Figure 6 illustrates the biosynthesis of NPs using phytochemical constituents extracted from plant sources.

AgNPs are the widely studied plant-based NPs because they are associated with potent antibacterial and antifungal activity. Various plant-based NPs with antimicrobial activity are mentioned in Table 8. AgNPs were synthesized using an aqueous extract derived from cell cultures of *Catharanthus roseus* (Apocynaceae) leaves, calli, and roots [205]. Several clinical pathogens, including *Candida albicans*, *Escherichia coli*, *Staphylococcus aureus*, *Bacillus subtilis*, and *Klebsiella pneumoniae* were the subjects of investigation. It was demonstrated that the stabilized AgNPs had the highest level of efficacy against all pathogens tested. There is optimism that plant-based NPs can be widely employed because of this alternative, rapid method to produce NPs with specific qualities that are effective, reasonably inexpensive, and ecologically benign [199,205]. Using their aqueous leaf extracts, *Senna siamea* (*Fabaceae*), *Crossopteryx febrifuga* (*Rubiaceae*), and *Brillantaisia owariensis* (=*B. patula*, *Acanthaceae*) were demonstrated to be effective in producing AgNPs in an environmentally friendly manner. When compared to their respective crude extracts and AgNO_3_, the AgNPs generated from them showed greater antibacterial efficacy against three bacterial pathogens that cause skin infections in humans. This suggests that biomolecules can enhance the biological activity of metal NPs [206].

When coupled with ciprofloxacin, AgNPs from the well-known medicinal plant *Fagonia indica* (Zygophyllaceae) showed superior efficacy against *Salmonella typhi*, *Citrobacter amalonaticus*, *E. coli*, and *Shigella sonnei*. Since “green” AgNPs and conventional antibiotics have a positive synergistic impact that is superior to that of AgNPs alone, it is important to take note of any potential interactions between antibiotics and AgNPs [211]. The most notable effect, for instance, was shown when amoxicillin and ecologically friendly bottom-up-produced AgNPs were tested against *Serratia marcescens*, demonstrating a 17.8-fold increase in the inhibitory zone. This demonstrates the synergistic boosting effect of amoxicillin and AgNPs capped with aqueous extracts from the leaves of *Urtica dioica* (Urticaceae) [207]. There have also been other documented interactions between antibiotics and plant-based NPs. AgNPs and maize leaf waste from a *Zea mays* substance (Poaceae), combined with kanamycin and rifampicin, for instance, were effective against five types of bacteria [208]. Similarly, AgNPs as well as extracts of the leaves of *Typha angustifolia* (Typhaceae), mixed with gentamicin, cefotaxime, or meropenem, were effective against *E. coli* and *Klebsiella pneumonia* [209]. Therefore, by reducing the toxicity and resistance, the use of antibiotics in conjunction with synthetic green metallic NPs seems to be a potentially effective way to tackle multidrug resistant organisms. The potential application of AgNPs as prospective antibacterial agents is further strengthened by the development of ecologically friendly methods for synthesis that take into account the need for safe solvents, as well as the agents that reduce and cap through these pathways [212].

Therefore, the development of a sustainable NP synthesis method utilizing plant extracts is warranted, as it would represent a significant advancement in the realm of applications for nanotechnology [205]. Albeit not the only bio-based metallic NPs having antibacterial properties, AgNPs have been the subject of the most investigation. Nickel NPs (NiNPs) derived from *Ocimum tenuiflorum* (=*O. sanctum*, Lamiaceae) leaf extract have also been shown to be more effective than the leaf extract alone and antibiotics as a disinfectant that works against Gram-positive bacteria (*Staphylococcus epidermidis*, *Bacillus subtilis*), Gram-negative bacteria (*K. pneumoniae*, *Salmonella typhi*, and *E. coli*), and fungi (*Candida albicans*, *C. tropicalis*, *Aspergillus fumigatus*, *A. clavatus*, and *A. niger*) [213]. TiO_2_ nanoparticles (NPs) exhibit greater biological activity than other oxides of metals, including the ability to interact with antibiotics in water, and they have antibacterial (and anticancer) properties. Additionally, TiO_2_ photoexcitation facilitates the horizontal transfer of resistance genes by phage transduction. Nevertheless, further research is required to assess the impact of NPs on the survival of bacteria and resistance genes in bacteria that are more resistant to NPs than those that are susceptible to NPs [214].

### 7.3. Nanoformulated Antibiotics

“Nano-antibiotics” are nanomaterials that exhibit antibacterial activity or improved antibiotic efficacy and safety [215]. They have numerous benefits over conventional antibacterial drugs, such as increased efficiency against drug-resistant bacteria, fewer side effects, and the ability to disrupt several cellular pathways [16]. Antimicrobial nanomaterials can break down biomolecules such as nucleic acids, fats, carbohydrates, lipids, and proteins, which alter enzymatic characteristics, prevent energy transfer, and damage the bacterial cell membrane. A broad spectrum of metal-based NPs have been shown to have antibacterial properties, such as copper, nitric oxide, aluminum, zinc oxide, silver, gold, and zinc. These NPs cause harm to the bacterial cell membrane, generate reactive oxygen species (ROS), obstruct intracellular enzyme activity or DNA synthesis, and disrupt the transmission of energy [216]. According to previous reports, ZnONPs are very efficient broad-spectrum antibacterial agents against bacterial species that are significant to medicine. ZnONPs exhibit antimicrobial properties through the oxidation of bacterial cell membrane proteins and lipids by ZnI_2_ ion-mediated ROS. This reaction leads to the leakage of the intracellular contents and ultimately the death of the cells of bacteria [217]. Experiments were conducted on the antibacterial activity of AgNPs produced by *Fusarium oxysporum* against drug-resistant pathogens, including *Klebsiella pneumoniae*, *Pseudomonas aeruginosa*, *Enterobacter* species, and *Escherichia coli*. The presence of NPs was shown to render the targeted bacteria sensitive to antibiotics [218].

**Figure 7:**
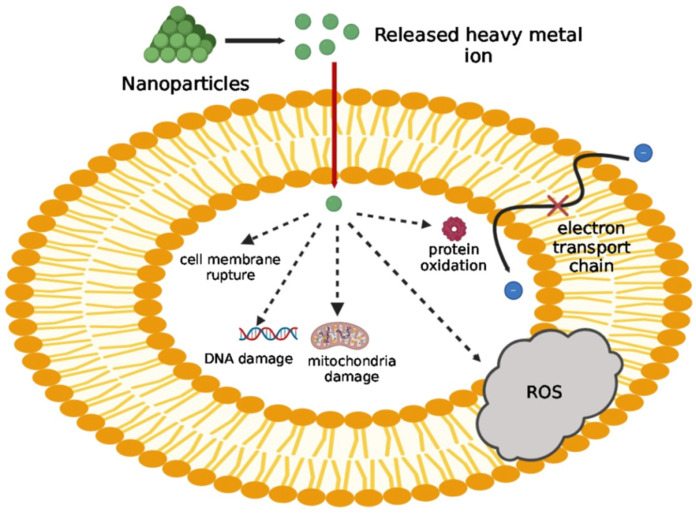
**Mechanisms of action of NPs (created in © BioRender—biorender.com).** NPs exert antibacterial action through mechanisms such as damaging bacterial cell membranes, generating ROS that disrupt intracellular processes like DNA synthesis and enzyme function, and interfering with bacterial energy metabolism. These actions collectively lead to bacterial cell death and enhance the efficacy of antimicrobial treatments.

A great deal of potential has been demonstrated for many NPs for the target-specific administration of antimicrobial drugs, especially antibiotics. Owing to their distinct physical and chemical characteristics, including their microscopic and accessible size, high surface-area-to-mass ratio, and strong interactions with microorganisms and host cells, nanomaterials have immense potential as platforms for the optimization of the therapeutic effects of antimicrobials. Similarly, nanocarriers offer a variety of benefits, such as improving the solubility of medications with low water solubility, extending the period of the drug’s half-life and systemic circulation, and slowing the drug’s release, all of which reduce the frequency and dosage of administration [219]. Furthermore, utilizing nano-vehicles to co-deliver various antimicrobial drugs might result in fewer systemic adverse effects than the focused administration of antibiotics alone [220]. Figure 7 illustrates the diverse mechanisms by which nanoparticles exert their antimicrobial effects.

### 7.4. Nano-Inhibitors of QS

Several nanomaterials have been discovered as potential nano-factories for the synthesis of antimicrobials that modify bacteria’s QS systems, rather than their viability. For instance, SLNs were employed to improve mucus penetration, pulmonary treatment, and anti-virulence effectiveness in *P. aeruginosa*, making them the very first in situ anti-QS nanomaterials developed recently [149]. This nanomaterial enhances its therapeutic efficacy by taking advantage of the unique characteristics of SLNs by integrating QSIs into SLNs. Initially, deeper delivery into the respiratory tract and increased interaction with *P. aeruginosa* populations living in the mucus layer are made possible by SLNs’ enhanced mucus penetration of SLNs. The pulmonary administration route enabled by SLNs ensures targeted delivery directly to the site of infection, maximizing the concentrations of QSIs at the site of infection, while reducing systemic side effects. The extended release of QSIs facilitated by SLNs enhances the anti-virulence activity of the nanomaterial by prolonging the suppression of QS signaling, thus reducing the virulence factors of *P. aeruginosa.* Overall, the synergistic combination of SLNs with QSIs not only enhances the pharmacokinetic properties and bioavailability of the therapeutic agents, but also augments their anti-virulence effects, offering a promising strategy to combat *P. aeruginosa* infections. Utilizing the metabolic products of a medicinal lichen (*Usnea longissima*), the authors’ group has recently created a unique herbo-metallic colloidal nanoformulation based on gold that efficiently attenuates *Streptococcus* mutans’ QS signaling [15]. The synergistic action of the metallic and herbal ingredients may enhance the antibacterial capabilities of the mixture. The purpose of the nanoformulation is to interfere with bacterial communication and possibly reduce *Streptococcus* mutans’ pathogenicity by weakening its QS signaling, which could potentially be used to prevent or treat dental caries. Fernandes et al. created biological nano-factories that were developed to modify the QS response in certain bacteria, including *Salmonella typhimurium* and *E. coli.* Engineered biological nano-factories are synthetic structures that resemble biological processes, most likely at the nanoscale level. Bacterial behavior, particularly pathogenicity and biofilm development, may be affected by the nano-factories’ propensity to obstruct communication. The researchers came to the conclusion that nano-factories are superior to typical QSIs in a number of ways, including better mucus and biofilm penetration [221].

### 7.5. Nano-Inhibitors of the QS System in Bacteria

Using hot-melt homogenization, QSI-loaded ultra-small solid–lipid (us-SL) NPs were synthesized using a variety of pharmaceutical-grade lipids. The extent of these NPs’ anti-virulence effectiveness against *Pseudomonas aeruginosa* was assessed by measuring the amount of pyocyanin produced. Compared to the free chemicals, us-SLNPs showed a seven-fold increase in anti-virulence activity. Remarkably, as nanocarriers, even unmodified SLNPs had intrinsic anti-virulence characteristics, which were ascribed to the anti-virulence qualities of the emulsifiers used in their preparation. This implies that the emulsifiers, pharmaceutical-grade lipids, and resultant NPs work in concert to increase the anti-virulence action of the product. These results highlight the possibility of using formulations based on NPs to greatly increase the effectiveness of anti-virulence treatments against *P. aeruginosa* infections [149]. AgCl-TiO_2_ (AT) NPs have been linked to improved food safety and the prevention of food spoiling. The ability of these NPs to prevent the synthesis of AHLs and violacein indicates their anti-QS action. TiO_2_ acts as a strong supporting matrix in the NPs, allowing silver to be released gradually and extending its residual activity. Additionally, although there is no negative effect on the proliferation of planktonic cells, zinc ions and ZnONPs have inhibitory effects on the release of virulence factors in *Pseudomonas aeruginosa*, including pyocyanin, PQS, pyochelin, and hemolysis [222]. This points to a synergistic interaction between the various constituents of these NPs, wherein the activity of zinc, TiO_2_, and silver ions together improves their overall anti-virulence characteristics, rendering them attractive candidates to combat bacterial infections and improve food safety standards. A new method of controlling membrane biofouling has been presented by the inventive use of immobilized QS molecule-degrading enzymes in a submerged membrane bioreactor for the treatment of wastewater. In particular, acylase was immobilized on a nanofiltration membrane. Acylase is an enzyme that breaks down AHLs, which are important signaling molecules in bacterial QS systems. Through a reduction in the release of exopolysaccharides, an essential component of bacterial biofilms, this targeted immobilization successfully hindered the formation of mature mushroom-like biofilms. The combination of membrane filtration technology and enzyme immobilization produces a synergistic effect because the membrane filtration system physically separates and removes the treated wastewater from the environment, while the enzyme activity directly targets AHLs, preventing bacterial communication and biofilm formation [223]. By targeting biofouling problems in wastewater treatment systems, this integrated strategy shows promise as a practical and long-term way to improve water quality and environmental health.

As opposed to having an immediate impact on the bacteria’s capacity to survive, nano-factories are thought to be tools to target their communication networks. This implies a focus on interfering with the QS process, which is how bacteria coordinate their behaviors according to the population density. Because nano-factories can hold payloads, such as fusion proteins and antibodies, QSIs can be successfully delivered to bacterial cells. This delivery method enables the modulation of bacterial QS signaling pathways. These nano-factories demonstrated a selective targeting capacity when added to mixed cultures of bacteria, specifically focusing on the appropriate bacteria in the mixture. Once they are attacked, nano-factories cause the bacterial population to mount a QS response. Apart from their function of focusing on certain populations of bacteria, nano-factories also possess the capacity to initiate communication among bacterial communities that may not otherwise interact with one another. This implies that there may be scope to improve our knowledge of and ability to manipulate interspecies bacterial interactions. ZnONP treatment increased the expression levels of transcriptional regulators (porin gene opdT and type III repressor ptrA) and a QS-related operon of *P. aeruginosa*, but decreased the level of the phenazine-related operon, according to a transcriptome analysis [222]. These results offer an innovative perspective that will ultimately be important for the creation of nano-anti-virulence medications. Table 9 illustrates a list of metallic and polymeric nanoformulations employed in the deactivation of QS across specific bacterial strains.

### 7.6. Green Nano-Inhibitors

A significant advancement in nanotechnology that will enable the use of NPs in nanomedicine is the creation of nontoxic synthesis techniques. Green chemistry is being used in bio-synthesized nanomaterials, which have recently gained a lot of interest because they are inexpensive, environmentally beneficial, and renewable. Two types of organic plant gum (ghatti and olibanum), which produce biogenic AgNPs, were compared for their antibacterial effects. The ghatti gum-synthesized AgNPs showed the strongest anti-biofilm activity against both Gram-negative and Gram-positive bacteria [232]. AgNPs that were synthesized biologically using *Bacillus licheniformis* precipitation had strong anti-biofilm activity against *S. epidermidis* and *P. aeruginosa* [233]. After 24 h of exposure, the bacteria showed >95% suppression of pre-existing biofilm development. According to the researchers, the reason that AgNPs hinder pre-existing biofilms might be due to the presence of water canals within the biofilm, which allow NP penetration. AgNPs may spread straight across water channels by penetrating the exopolysaccharide layer.

AuNPs were coated with active AHL lactonase (AiiA) proteins that were biologically synthesized from *B. licheniformis*. These nanoparticles disrupted the structures of biofilms and prevented Proteus species from producing exopolysaccharides [234]. The researchers postulated that the AiiA protein and AuNPs’ synergistic interactions may accelerate QS molecule breakdown and hence prevent the development of biofilms. 

Using extracts from fresh *Cymbopogan citratus* (lemongrass) leaves, a more environmentally friendly approach to producing AgNPs with extended durability has been developed [235]. These NPs limited the formation of biofilms by exhibiting enhanced QQ activity towards *S. aureus*. When *C. violaceum* CV026 was cultivated in an exogenous AHL environment, AgNPs from the aqueous extracts of the brown seaweed Sargassum polyphyllum produced a decrease in purple pigment, indicating QSI. Moreover, *P. aeruginosa* demonstrated the QQ action of AgNPs by losing its capacity for swarming movement while maintaining bacterial growth [236]. *P. aeruginosa* RhlI/RhlR mutants were previously shown by Kohler et al. to be completely incapable of swarming, which explains the participation of the *P. aeruginosa*’s cell-to-cell signaling network under NP activity [237].

## 8. Discussion

Bacteria are foundational unicellular organisms that possess remarkable capabilities for independent growth, division, and responsiveness to environmental cues. Despite their individual autonomy, bacteria exhibit coordinated behavior through mechanisms such as QS, which allows them to communicate and collectively regulate gene expression based on population density changes. QS is critical in synthesizing virulence factors for the pathogenesis and development of biofilms—cohesive bacterial communities adhering to surfaces. However, the increasing resistance of bacteria to conventional antibiotics poses a major obstacle in medical practice, where new drugs are exhibiting reduced efficacy against certain strains. This demands the exploration of novel antimicrobial therapies that target specific bacterial components responsible for virulence, rather than simply inhibiting growth. QQ is a potential strategy that involves blocking QS pathways to reduce the synthesis of virulence factors and biofilm development. QQ can target various points in the QS pathway, offering the potential for the effective control of bacterial behavior. Small substances from natural sources like marine organisms, animals, plants, and fungi are being investigated as QSIs, alongside macromolecules like acylases, oxidoreductases, and AHL lactonases. Through their interference with QS signaling, these chemicals demonstrate potential in reducing bacterial virulence and biofilm formation. In the realm of antibacterial therapy, nanotechnology emerges as a game-changing strategy. Nanomedicine offers the precise delivery and targeting of antimicrobial agents, enhancing their efficacy while minimizing their toxicity. NPs, characterized by their small size, are integral to nanotechnology-based drug delivery. Organic NPs (e.g., liposomes and polymeric NPs) offer versatility in handling different drugs but face challenges such as instability under certain conditions. Inorganic NPs (e.g., AgNPs and AuNPs) with unique physicochemical properties show promise as antibacterial agents, overcoming the constraints related to traditional antibiotics. Nanoantibiotics or nanomaterials exhibiting antibacterial properties or enhancing antibiotic efficacy are gaining traction because of their ability to combat drug-resistant bacteria with fewer side effects. Nanoinhibitors that specifically target bacterial QS systems are currently under development. These nanoinhibitors disrupt bacterial communication rather than viability, showing the potential to effectively combat bacterial virulence. Green nanoinhibitors, synthesized through eco-friendly methods such as green chemistry, offer an environmentally beneficial approach for the development of antimicrobial nanomaterials. Incorporating plant-based NPs into nanoantibiotics further expands the arsenal against bacterial infections, capitalizing on naturally occurring antimicrobial substances. Plant-derived NPs demonstrate varied forms and sizes are influenced by their source location, and present intriguing possibilities for the development of effective nanoantibiotics. This convergence of innovative nanotechnologies with natural antimicrobial resources is a promising frontier in the fight against multidrug-resistant bacteria, aiming for sustainable and effective antimicrobial therapies.

## 9. Conclusions and Future Perspectives

In conclusion, the investigation of compounds that target bacterial QS pathways has gained traction in recent years as a potentially effective antimicrobial intervention strategy. However, traditional QSIs face challenges, such as limited bioavailability and inefficient penetration in vivo. The development of nanofactories as a new class of antibacterial compounds and delivery systems holds promise, leveraging their unique physicochemical properties for enhanced delivery and efficacy.

When formulating antibacterial treatments, it is vital to create nanocarriers that stabilize the active components and enhance their delivery to sites of infection. Nanomedicine has garnered growing interest as a means of targeting bacteria or delivering drugs, with NPs loaded with antibiotics presenting various benefits over traditional formulations. These include enhanced stability, controlled antibiotic release, targeted delivery, and improved bioavailability. Although NPs have shown potential as carriers for QSIs and conventional antimicrobials, comprehensive data on their clinical applications and toxicity profiles are still needed.

To bridge this gap, we require a multifaceted approach that encompasses both theoretical exploration and clinical validation. Challenges, such as the long-term toxicological effects of NPs and the imperative need for interdisciplinary collaboration, persist, underscoring the need for concerted efforts across scientific disciplines and regulatory frameworks. The escalating threat of antibiotic resistance emphasizes the urgent need for innovative antimicrobial strategies. Metallic NPs exhibit potent antimicrobial properties; however, elucidating their precise biocidal mechanisms and addressing issues of standardization are imperative for rational antimicrobial therapy.

The multitude of resistant microbial mechanisms has undoubtedly presented significant hurdles in researching and resolving resistance concerns. As a result, understanding the development of bacteria’s primary resistance mechanisms is critical for microbial ailment control and prevention. At present, QS has great potential as a therapeutic target for bacterial diseases, as demonstrated by the variety of therapeutic approaches that have been employed, such as receptor deletion, antibody-mediated signaling blockage, and co-administration with antibiotics.

Receptor deactivation in QS signaling is a helpful strategy to reduce infection and bacterial pathogenicity. Studies have demonstrated that flavonoids can attach to QS receptors and significantly reduce the expression of virulence genes in *Pseudomonas aeruginosa* [238]. AHL compounds have been produced by Geske et al. that can bind to the LuxR, TraR, and LasR receptors in *Pseudomonas aeruginosa*, *Agrobacterium tumefaciens*, and *Vibrio fischeri*, respectively [239]. However, receptor inhibitors are not as popular in the treatment of bacterial infections because of their instability and destruction in alkaline settings.

Enzymes that break down QS signals can effectively stop the bacteria from “communicating” with one another, without placing he bacterium under any form of selection pressure. Employing AHL lactonases to boost the antibiotic sensitivity and inhibit biofilm formation is a therapeutic technique that aims to modulate bacterial behavior by disrupting QS. Furthermore, using antibodies that are monoclonal to target and disrupt QS or QS signals provides a therapeutic method with high potential. These antibodies preferably bind to QS signaling molecules or hinder their function. They impair bacterial communication, resulting in reduced virulence component production and bacterial pathogenicity.

Currently, the most successful therapeutic approach for treating bacterial illnesses is the combination of antibiotic usage with an anti-QS drug. Gallocatechin-3-gallate and caffeic acid are examples of anti-QS compounds that, when combined with doxycycline, ciprofloxacin, or gentamicin, enhance their therapeutic benefits in *M. pneumoniae* infections [240]. N-(2-pyrimidyl) butylamine has been shown to enhance the antibacterial activity of tobramycin, ciprofloxacin, and colistin against *Pseudomonas aeruginosa* [241].

This novel non-antibiotic treatment has been widely applied recently and is capable of inhibiting the synthesis of harmful genes, preventing infection, and reducing the risk of drug-resistant bacterial cells. Numerous investigations have presented a wide range of anti-QS compounds that can both reduce the pathological damage in different animal infection models and regulate the pathogenic phenotypes of the majority of bacteria. However, since most anti-QS drugs are currently in the preclinical stage, more human clinical trials are required to assess their practicality.

Ultimately, the integration of nanotechnology with antimicrobial research offers hope in combating infectious diseases. By leveraging nanoengineering to optimize drug delivery and mitigate adverse effects, the trajectory towards efficacious nanomedicines is promising. However, addressing existing challenges and translating promising findings into clinical applications are essential to realize the full potential of nanotechnology in combating bacterial infections.

## Figures and Tables

**Table 1:** Summary of QSIs from various animal sources and their activities.

Source	Inhibitor	Target QS Signals	Activity	Reference
Porcine Kidney	Acylase I	C6HSL, 3OC12HSL	Deactivates C6HSL and 3OC12HSL; does not affect N-Butanoyl-L-Homoserine Lactone (C4HSL); moderate biofilm inhibition	[23,53]
Mammals (e.g., Bovine, Goat, Rabbit, Horse, Mouse)	Paraoxonases (PONs)	Esters and lactones	Hydrolyze esters and lactones; differ from prokaryotic lactonases; require calcium ions	[54,55]
Human Epithelial Cells	Endogenous Enzymes	AHLs	Deactivate AHLs produced by *Pseudomonas aeruginosa*; efficacy varies with acyl chain length	[56]
Serum (various mammals)	Natural Components	3OC12HSL	Deactivate 3OC12HSL	[57]
Food Products	AI-2 Inhibitors	AI-2	May contain compounds mimicking AI-2 activity	[58,59,60]
Ground Beef	Extracts	AI-2	Suppress AI-2-mediated bioluminescence in *Vibrio harveyi*; alter virulence gene expression	[61]

**Table 2:** Summary of QSIs derived from various fungi.

Source	QSI	Target Organism	QS Activity	Reference
*Tremella fuciformis*	Fruiting bodies	*Chromobacterium violaceum*	Inhibition of violacein production	[66]
*Auricularia auricular*	Natural pigments	*Chromobacterium violaceum*	Inhibition of violacein production	[65]
*Penicillium*	Patulin and penicillic acid	*Pseudomonas aeruginosa*	Inhibition of biofilm formation	[63]

**Table 3:** Summary of QSIs derived from various marine organisms.

Source	QS Inhibitor	Target Organism	Activity	Reference
*Ahnfeltiopsis flabelliformis*	α-D-galactopyranosyl-(1 → 2)-glycerol(floridoside), betonicine, and isoethionic acid	*Agrobacterium tumefaciens*	Inhibits AHL-mediated QS	[81]
*Chlamydomonas reinhardtii*	Unidentified AHL mimics	*Escherichia coli*	Inhibits bioluminescence	[59]
*Delisea pulchra*	Halogenated furanone	*E. coli*	Inhibits biofilm formation and swarming	[82]
	AI-2 signaling system	*Proteus mirabilis*	Inhibits swarming motility	[83]
		*Pseudomonas aeruginosa*	Inhibits biofilm formation	[84]
		*Serratia liquefaciens*	Inhibits swarming motility	[85]
		*Vibrio fischeri*	Inhibits bioluminescence	[85]
		*Vibrio harveyi*	Inhibits toxin production and luminescence	[86]
			Inhibits biofilm formation	[87]
			Inhibits bioluminescence	[85]
*Laminaria digitata*	Oxidized halogen HOBr	*Chromobacterium violaceum* CV026	Specific inhibition of 3-oxo-acyl HSLs	[88]
*Blennothrix cantharidosmum*	Tumonoic acids	*Vibrio harveyi* BB120	Suppresses bioluminescence without affecting growth	[67]
*Lyngbya majuscula*	Lyngbyoic acid, malyngolide, 8-epi-malyngamide C, lyngbic acid	*Pseudomonas aeruginosa*	Inhibits LasR response to 3OC12-HSL	[68]
*Halobacillus salinus*	N-(2-phenylethyl)-isobutyramide, 3-methyl-N-(2-phenylethyl)-butyramide	*Chromobacterium violaceum* CV026	Inhibits violacein biosynthesis	[69]

**Table 4:** QSIs derived from various plant sources.

Source	QSI	Effective against	Anti-QS Activity	Reference
*Allium sativum*	extracts	*A. tumefaciens* NTL4; *C. violaceum*; *P. aeruginosa*	Supresses β-galactosidase and violacein, alginate, and elastase production; biofilm formation; fluorescence	[105,106,107,108]
*Alyssum maritimum*	leaf	*C. violaceum* CV0blu	Slight QS inhibition	[109]
*Ananas comosus*	-	*C. violaceum*; *P. aeruginosa* PAO1	Inhibits violacein production, pyocyanin, staphylolytic protease, elastase, and biofilm formation	[103]
*Arabidopsis* exudate	γ-hydroxybutyrate (GHB)	*A. tumefaciens*	Inhibits AHL signaling	[102]
Blueberry	extracts	*C. violaceum*	Inhibits violacein production	[90]
*Brassica napus*	leaf	*C. violaceum* CV0blu	Slight QS inhibition	[109]
*Brassica oleracea*	extracts	*C. violaceum*	Inhibits violacein production	[90]
*Cinnamomum zeylanicum*	cinnamaldehyde	*P. aeruginosa*; *E. coli*; *V. harveyi*	Inhibits biofilm formation and AHL- and AI-2-mediated QS	[110]
Grape	extracts	*C. violaceum*	Inhibits violacein production	[90]
Grapefruit juice	furocoumarins	*E. coli;P. aeruginosa*; *Salmonella typhimurium*	Inhibits biofilm formation	[96]
*Lotus corniculatus*	GHB	*A. tumefaciens* NTLR4; *C. violaceum* CV026	Inhibits beta-galactosidase and violacein production	[111]
*Manilkara zapota*	-	*C. violaceum*; *P. aeruginosa* PAO1	Inhibits violacein production and pyocyanin, staphylolytic protease, elastase, and biofilm formation	[103]
*Musa paradiciaca*	-	*C. violaceum*; *P. aeruginosa* PAO1	Inhibits violacein production and pyocyanin, staphylolytic protease, elastase, and biofilm formation	[103]
*Medicago sativa*	L-Canavanine	*C. violaceum*; *Sinorhizobium meliloti*	Inhibits violacein production and Exopolysaccharide II (EPSII)	[93]
*Ocimum basilicum*	rosamarinic acid	*P. aeruginosa*	Inhibits protease and elastase production; biofilm formation; and virulence factor production	[112]
*Ocimum sanctum*	-	*C. violaceum*	Inhibits violacein production	[103]
*Passiflora incarnata*	leaf	*C. violaceum* CV0blu	Inhibits violacein production	[109]
*Pisum sativum*	seedlings, leaves, roots	*C. violaceum* CV026	Inhibits C4HSL-inducible protease and N-acetylglucosaminidase; violacein production; and swarming activity	[113]
Raspberry	extracts	*C. violaceum*	Inhibits violacein production	[90]
*Romneya trichoclyx*	leaf	*C. violaceum* CV0blu	Slight QS inhibition	[109]
*Ruta graveolens*	leaf	*C. violaceum* CV0blu	Slight QS inhibition	[109]
*Scorzonera sandrasica*	extract	*C. Violaceum* D. ATCC12472; *Erwinia caratovora*	Inhibits violacein production and carbapenem antibiotic production	[113]
Squash exudate	GHB	*A. tumefaciens*	Inhibits AHL signaling	[102]
Tomato seedling exudate	GHB	*A. tumefaciens*	Inhibits AHL signaling	[102]
*Vanilla planifolia*	extract	*C. violaceum* CV026	Inhibits violacein production	[114]
*Combretum albiflorum*	bark	*P. aeruginosa*	Inhibits biofilm formation	[99]

**Table 6:** Overview of organic NPs.

Types of NPs	Description	Advantages	Limitations	Toxicity	References
SLNs	Lipid-containing colloidal carriers, solid at body temperature, can deliver drugs like antibiotics precisely to infection sites and enhance efficacy against bacterial biofilms.	Less toxic and more stable than synthetic polymers, with controlled release properties. They enhance CNS drug targeting, support various administration routes, and effectively reduce biofilm biomass with antibiotics or phytochemicals.	May pose local toxicity risks, burst release issues, and formulation complexities that can affect stability, efficacy, and large-scale production. Effectiveness can vary with biofilm type or bacterial strain.	Generally less toxic due to the use of biological lipids, but may still cause local toxicity or hypersensitivity depending on the formulation, dose, or bioactive components.	[160,161,162]
Polymeric Micelles	Colloidal particles formed from block copolymers at specific micellization temperatures and concentrations. They have a hydrophilic outer shell and a hydrophobic core. Micelle stability is influenced by polymer chain length and drug charge density.	Low toxicity, enhance permeability and retention, and have fewer biocompatibility issues, making them ideal for extended circulation, gene transfer, imaging, and targeted drug delivery.	Poor drug loading and integration stability can cause early drug release, and the micelle size is constrained by the polymer’s chemical structure.	Generally low toxicity; micelles break down into harmless individual polymer chains and are easily removed.	[163,164]
Polymeric NPs	Composed of natural or synthetic polymers with hydrophilic and hydrophobic components. They protect and control the release of drugs and can be produced from polymers like PLGA, PLA, PCL, PCA, alginate, albumin, and chitosan.	Enhance drug stability, bioavailability, and solubility; provide sustained release; and are effective against bacterial biofilms and various pathogens. Chitosan is especially valued for its mucoadhesive and antimicrobial properties.	Limited capacity to modify dosage. Particle agglomeration can hinder physical manipulation of forms. Effectiveness varies with polymer choice and synthesis method, affecting shape, size, and surface properties.	Not specifically mentioned in the text, but polymeric NPs are generally considered to have low toxicity due to their design for targeted drug delivery.	[165,166,167]
Liposomes	Spherical vesicles with phospholipid bilayers, ranging from 20 nm (small unilamellar) to >100 nm (multilamellar). They fuse with bacterial membranes to release contents and can encapsulate both hydrophilic and hydrophobic drugs.	Enhance antiviral and antimicrobial efficacy, can be modified for better stability and targeting, and effectively encapsulate a wide range of drugs. Also effective against biofilm-associated infections by penetrating EPS and improving antibiotic delivery.	Potential for drug encapsulation leakage. Subject to absorption and elimination by the reticuloendothelial system (RES).	Generally considered safe but may vary depending on the formulation and application. There is no specific toxicity noted in the provided text.	[168,169]

**Table 7:** Overview of inorganic NPs.

Types of NPs	Description	Advantages	Limitations	Toxicity	References
Silver nanoparticles (AgNPs)	AgNPs have a broad antibacterial spectrum and strong bactericidal properties, effectively targeting a wide range of microorganisms by damaging cell membranes and increasing oxidative stress.	Effective against both Gram-positive and Gram-negative bacteria. Better antibacterial action than some antibiotics. Used in various biomedical applications, including biosensors, coatings, and medical devices. Proven potent anti-biofilm activity in several studies.	Size, shape, and capping agent affect antibacterial effectiveness. Potential issues with poor drug loading and stability. Limited information on long-term effectiveness and application stability.	Potential cytotoxicity and allergic reactions. Risk of accumulation, leading to skin discoloration and problems with the nervous system, kidneys, or liver. Toxicity largely due to the cytotoxic effects of silver ions (Ag^+^).	[175,176,177]
Gold NPs (AuNP)	AuNPs have strong near-infrared (NIR) absorption and versatile properties, making them valuable in biomedical applications for detection of proteins, DNA, and bacteria, with potential for enhanced visibility and detection.	Strong light-scattering and optical properties. Effective for drug delivery, biomarker detection, and imaging. Can be modified with various ligands for enhanced functionality.	Low biocompatibility and weak optical signals. Limited efficacy in tumor targeting.	Risks of acute and chronic toxicity due to non-biodegradability.	[178,179,180]
Iron Oxide NPs (IONPs)	Widely used inorganic compounds with diameters ranging from 1 to 100 nm. They exhibit superparamagnetic behavior when their particle size is reduced to less than 30 nm at room temperature.	Effective in bacterial detection and targeted drug delivery. Useful in MRI and biosensing. Improved stability and biocompatibility with silica coating. ZnO and other MNPs have shown potent anti-biofilm activity and disruption of biofilm matrices.	Naked IONPs can agglomerate and lose magnetism due to airborne oxidation. Challenges in maintaining dispersibility and stability.	Potential toxicity from uncoated IONPs and high chemical activity. ZnO NPs can have varying degrees of toxicity depending on their formulation and application.	[181,182,183]
Quantum Dots (QDs)	Fluorescent nanoparticles, often produced from zinc or cadmium, used in bacterial detection and biological applications. QDs are typically coated with hydrophilic substances like PEG to improve water solubility and prevent aggregation.	Enhanced detection performance with long observation times. Versatile in tracking, drug delivery, and biological research. Small size and flexible surface modification for diverse applications.	Limited biodegradability and potential difficulty in diffusing across biological membranes. Limited transmittance in some cases may affect visibility.	Potential cytotoxicity and long half-life. Concerns about safety due to low biodegradability.	[184,185,186]

**Table 8:** Plant-based NPs with antimicrobial activity.

Plant (Family)	Plant Part	NPs	Antibiotics	Microorganism	Ref.
*Dioscorea* *bulbifera* (Dioscoreaceae)	Tuber	AgNPs	Piperacillin, erythromycin, chloramphenicol, vancomycin, streptomycin	*Acinetobacter* *baumannii,* *Pseudomonas* *aeruginosa,* *E. coli*	[204]
*Curcuma longa* (Zingiberaceae)	Tuber	AgNPs	n.c	*Escherichia coli* BL-21 strain	[32]
*Brillantaisia* *Owariensis* (Acanthaceae), *Crossopteryx* *febrifuga* (Rubiaceae), *Senna siamea* (Fabaceae)	Aqueous leaf extracts	AgNPs	n.c	Gram (+): *S. aureus* Gram (−): *E. coli*, *P. aeruginosa*	[206]
*Catharanthus roseus* (Apocynaceae)	Cell cultures from leaves, calli, roots	AgNPs	n.c	*Bacillus subtilis*, *Staphylococcus* *aureus*, *E. coli*, *Klebsiella* *pneumoniae*, *Candida albicans*	[3]
*Urtica dioica* (Urticaceae)	Aqueous leaf extracts	AgNPs	Amikacin, kanamycin, tetracycline, cefotaxime, amoxicillin, ampicillin, cefepime, vancomycin, streptomycin	Gram (+): *Bacillus cereus*, *B. subtilis*, *S. aureus*, *Staphylococcus* *epidermidis* Gram (−): *E. coli*, *Klebsiella* *pneumoniae*, *Serratia* *marcescens*, *Salmonella* *typhimurium*	[207]
*Fagonia* *Indica* (Zygophyllaceae)	Callus, Cell cultures	AgNPs	Ciprofloxacin	*E. coli*, *Citrobacter* *amalonaticus*, *Shigella sonnei*, *Salmonella typhi*	[5]
*Zea mays* (Poaceae)	Corn leaf waste extract	AgNPs	Kanamycin, rifampicin	*B. cereus*, *Listeria* *monocytogenes*, *S. aureus*, *E. coli*, *S. typhimurium*	[208]
*Ocimum* *tenuiflorum*	Leaf extract	NiNPs	n.c	Gram (−): *Klebsiella* *pneumoniae*, *Salmonella* *typhi*, and *E. coli* Gram (+): *Staphylococcus* *epidermidis*, *Bacillus subtilis* Fungi: *Candida albicans*, *C. tropicalis*, *Aspergillus* *fumigatus*, *A. clavatus*, and *A. niger*	[7]
*Typha angustifolia* (Typhaceae)	Leaf extract	AgNPs	n.c	*E. coli* and *K. pneumoniae*	[209]
*Piper guineense* (Piperaceae)	Aqueous leaf extracts	AuNPs	n.c	*S. aureus*, *Streptococcus* *pyogenes*	[37]
*Phyllanthus* *Reticulatus* (Phyllanthaceae), *Erigeron* *bonariensis* (=Conyza bonariensis) (Asteraceae)	Leaf extract	CuONPs	n.c	*E. coli*	[210]

n.c: not combined with antibiotics.

**Table 9:** Metallic and polymeric nanostructures utilized in suppressing QS among bacteria.

NP Type	Antibacterial Activity	References
Finer spike-like ZnONPs (diameter range 40–130 nm)	ZnONPs demonstrated a reduction of 89% and 85% in the swarming motility and production of pyocyanin, respectively, in *Pseudomonas aeruginosa* PAO1.	[224]
CuNPs in the size range of 66–69 nm	The minimum concentrations required to inhibit biofilm formation were 8 ppm for isoquercetin/CuNPs and 4 ppm for cassinopin/CuNPs against *Pseudomonas aeruginosa*.	[225]
Spherical AgNPs (diameter range 20–40 nm)	Diminished levels of rhlR gene expression in *Pseudomonas aeruginosa* strains that exhibit resistance to drugs.	[226]
The antibiotic kaempferol was encapsulated within chitosan/sodium tripolyphosphate (TPP) NPs, achieving encapsulation and loading efficiencies of 93% and 78%, respectively.	The synthesis of violacein by the *C. violaceum* CV026 strain led to inhibition rates of 33.92% and 76.21% at storage durations corresponding to T3 (after thirty days) and T0 (immediately after nanoparticle preparation), respectively.	[227]
AgNPs (mean range 5–30 nm) with agglomerated, spherical, and crystalline shapes	At a concentration of 25 µg/mL, AgNPs demonstrated significant reductions in the expression levels of various genes in *P. aeruginosa*. Specifically, lasR, rhlR, fabH2, lasI, and rhlI were downregulated by 51%, 36%, 81%, 71%, and 63%, respectively.	[228]
PAA-coated CuNPs (diameter 66–150 nm and zeta potential 13 mV)	The expression of the ppyR gene product, the primary regulatory protein of the Psl operon, exhibited an approximately 2.7-fold decrease when exposed to PAA-CuNPs, in contrast to Cu^2+^.	[229]
Chitosan-conjugated polymeric NPs incorporating EuNPs and myristyl myristate nanostructured lipid carriers (NLCs).	The prevention of swarming motility in *Pseudomonas aeruginosa* and *Staphylococcus aureus* was observed at a concentration of 50 μM, persisting for up to seven days.	[230]
ZnONPs (30 nm, spherical)	Suppression of violacein synthesis in *C. violaceum* CV026 and *Chromobacterium violaceum* ATCC 12472 within the concentration bracket of 100–250 mg/mL.	[231]
